# Lower functional hippocampal redundancy in mild cognitive impairment

**DOI:** 10.1038/s41398-020-01166-w

**Published:** 2021-01-18

**Authors:** Stephanie Langella, Muhammad Usman Sadiq, Peter J. Mucha, Kelly S. Giovanello, Eran Dayan, for the Alzheimer’s Disease Neuroimaging Initiative

**Affiliations:** 1 Department of Psychology and Neuroscience, University of North Carolina at Chapel Hill, Chapel Hill, NC USA; 2 Biomedical Research Imaging Center, University of North Carolina at Chapel Hill, Chapel Hill, NC USA; 3 Department of Mathematics, University of North Carolina at Chapel Hill, Chapel Hill, NC USA; 4 Department of Applied Physical Sciences, University of North Carolina at Chapel Hill, Chapel Hill, NC USA; 5 Department of Radiology, University of North Carolina at Chapel Hill, Chapel Hill, NC USA

**Keywords:** Neuroscience, Diseases

## Abstract

With an increasing prevalence of mild cognitive impairment (MCI) and Alzheimer’s disease (AD) in response to an aging population, it is critical to identify and understand neuroprotective mechanisms against cognitive decline. One potential mechanism is redundancy: the existence of duplicate elements within a system that provide alternative functionality in case of failure. As the hippocampus is one of the earliest sites affected by AD pathology, we hypothesized that functional hippocampal redundancy is protective against cognitive decline. We compared hippocampal functional redundancy derived from resting-state functional MRI networks in cognitively normal older adults, with individuals with early and late MCI, as well as the relationship between redundancy and cognition. Posterior hippocampal redundancy was reduced between cognitively normal and MCI groups, plateauing across early and late MCI. Higher hippocampal redundancy was related to better memory performance only for cognitively normal individuals. Critically, functional hippocampal redundancy did not come at the expense of network efficiency. Our results provide support that hippocampal redundancy protects against cognitive decline in aging.

## Introduction

Alzheimer’s disease (AD) is a neurodegenerative disease that poses a significant public health concern, with dementia constituting the fifth leading cause of death worldwide^1^. AD is characterized by the accumulation of amyloid β-plaques and neurofibrillary tau-tangles, which disrupt neural communication and contribute to functional and structural changes across the brain^2^. These pathologies aggregate during healthy aging and continue into mild cognitive impairment (MCI), regarded as a precursor stage to AD^3^. Although individuals diagnosed with MCI are more likely to later progress to AD, there is considerable variability in individual trajectories, with conversion estimates ranging from 8% to 25%^4^. In addition, the prevalence of biologically defined AD (diagnosed post-mortem) is up to three times higher than clinically defined AD, illustrating that a high proportion of older adults are presenting normal cognitive function, despite extensive neural pathology^5^. This suggests that in certain individuals, neuroprotective mechanisms allow the brain to cope with early neurodegeneration and retain normal cognitive function. With an increasing aging population, it is critical to identify the mechanisms that mitigate cognitive decline, yet these mechanisms are currently not well understood.

The general notion of reserve has been introduced to refer to the difference between the extent of brain damage and its outward presentation (clinically or cognitively)^6–8^. Thus, individuals with more reserve exhibit a resilience to or temperance of physical brain damage^6–8^. Reserve mechanisms in the brain are difficult to quantify. One potential quantifiable reserve mechanism is redundancy: the existence of duplicate elements within a system that provide alternative functionality in case of failure^9,10^. This design principle is abundant in engineering fields, where redundant elements protect a design from total failure in the event of malfunction of a specific element^11^. Redundancy exists in biological systems as well, with examples in genetic structures and cells, up to the level of whole organs^10,12,13^. For example, recent work has demonstrated that redundant elements are effective at preserving functioning in the event of gene deletions^10^ and providing robustness in neural networks^13^. As physical redundancy is not a requirement to support informational or functional redundancy^14^, functional redundancy can be calculated from a graph-based representation of functional brain networks^15,16^. Numerous studies have derived graph-based measures and topological properties from resting-state functional magnetic resonance imaging (rs-fMRI) data, in which systems are represented as a collection of nodes (brain regions) and edges (correlated time-series data)^17–19^. This approach has recently been employed to analyze functional redundancy in young adults, quantified as the sum of direct and indirect paths between any pair of nodes^15,16^. Similar approaches have been used to quantify redundancy in other biological networks, leading to the consideration that path redundancy (the presence of multiple paths between a pair of nodes) is an important contributor to robustness of cellular networks^20^. However, although the role of redundancy in neuroprotection has been postulated before^12,21^, it has not yet been formally quantified to date for studying neuroprotection in neurodegenerative diseases.

**Fig. 1:**
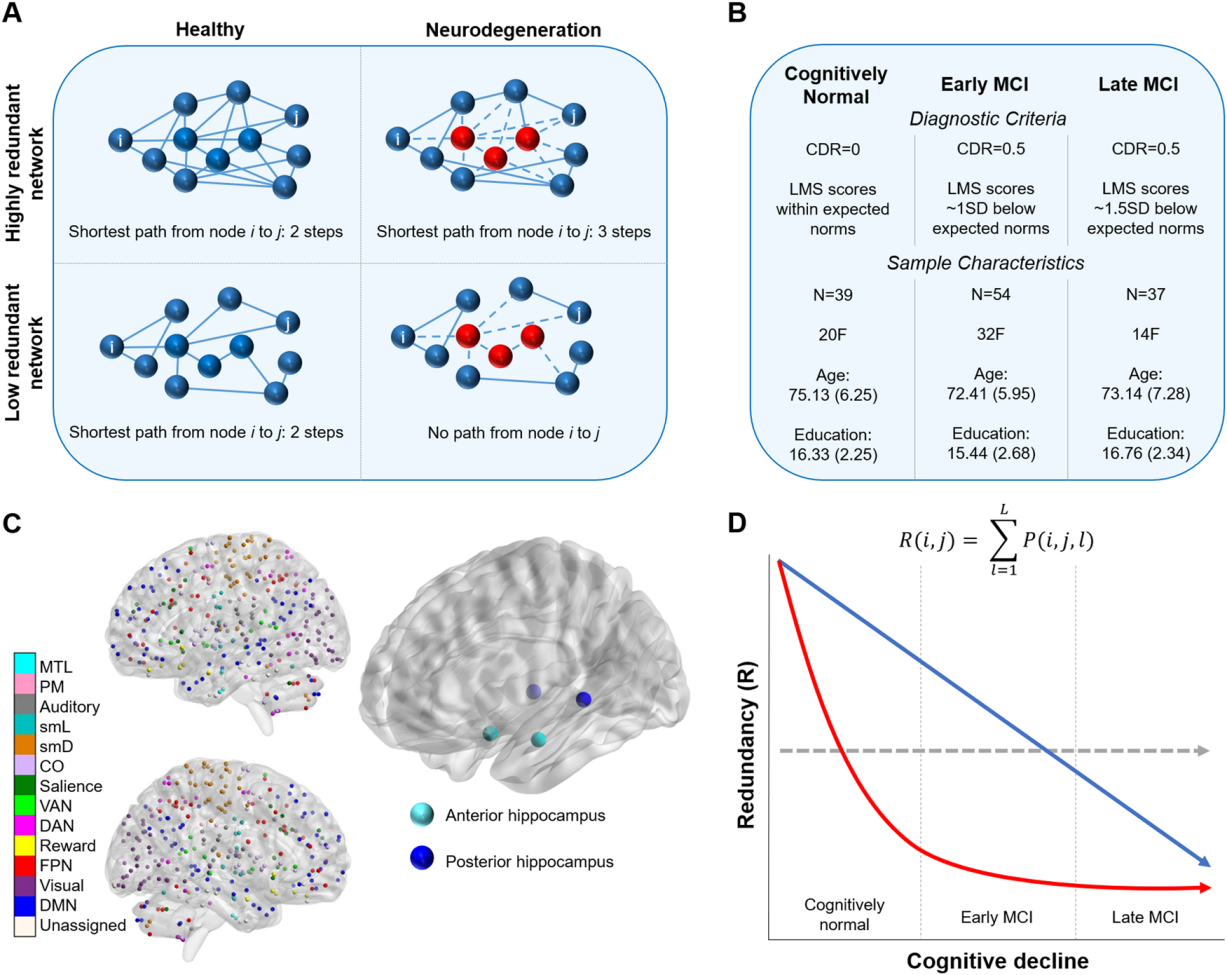
Study design and hypotheses. **A** Examples of networks with high and low redundancy. The shortest path between nodes *i, j* do not differ between high and low redundancy in a healthy state. In the case of neurodegeneration (red nodes), a highly redundant network retains a path between nodes *i, j*, whereas there are no paths between nodes *i, j* in a low redundancy network. **B** Sample characteristics of the included subjects. **C** Representation of the functional parcellation used in the current study made up of 300 nodes representing cortical, subcortical, and cerebellar regions^26^. The four hippocampal nodes are shown next to the full atlas parcellation, representing anterior (cyan) and posterior (blue) hippocampus. **D** Three hypothesized relationships between redundancy and cognitive decline. There could be no relationship between redundancy and cognitive decline (gray line), a linear relationship such that redundancy declines linearly across healthy aging, early MCI, and late MCI states (blue line), or a nonlinear relationship, such that redundancy declines between healthy aging and MCI, but plateaus in MCI (red line). Redundancy equation presented above hypothesized relationships, in which redundancy, *R*, of node pair *i,j* is represented by the sum of all paths between *i,j* at length *l*, up to a maximum path length, *L*.

It remains unknown, therefore, if redundancy is neuroprotective against age-related cognitive decline. A plausible site where neuroprotective functional redundancy may be detected is the hippocampus. This medial temporal lobe structure, critical for memory processes, is among the earliest sites affected by AD pathology^22^. Functional and structural alterations in the hippocampus are early and precede the onset of AD^5^, and MCI is characterized by declines in memory and hippocampal functioning^23^. We thus reasoned that hippocampal functional redundancy may serve as a neuroprotective mechanism to outward clinical presentation of MCI, such that in a redundant network, communication could continue even in the presence of neurodegeneration of a node (e.g., hippocampus) (Fig. 1A). Conversely, communication within a less redundant network should be severely disrupted in the presence of neurodegeneration^10^ (Fig. 1A).

The current study investigates functional redundancy in 130 cognitively normal (CN), early MCI (eMCI), and late MCI (lMCI) older adults (Fig. 1B) across four anterior and posterior hippocampal nodes (Fig. 1C), to elucidate how functional redundancy is associated with healthy (or asymptomatic) and pathological aging. Three potential relationships between redundancy and cognitive status were considered as follows: (a) no relationship between redundancy and cognitive status (i.e., redundancy is not a neuroprotective mechanism), (b) redundancy declines linearly with cognitive status (i.e., redundancy is protective in both healthy and pathological aging), or (c) redundancy declines from healthy to pathological aging, but plateaus upon reaching MCI (i.e., redundancy is neuroprotective in healthy or asymptomatic aging, but ceases to offer functional benefits upon appearance of neurodegeneration) (Fig. 1D). We hypothesize that functional redundancy will be related to diagnosis, such that CN individuals will have greater redundancy than those with MCI. Further, if redundancy acts as a neuroprotective mechanism, then we hypothesize that higher redundancy will be related to better cognitive performance.

## Materials and methods

### Dataset

Data were obtained from the Alzheimer’s Disease Neuroimaging Initiative (ADNI) database (adni.loni.usc.edu), a longitudinal multi-site study launched in 2003 and led by Principal Investigator Michael W. Weiner, MD. For up-to-date information, see www.adni-info.org. Subjects were participants of the ADNIGO/2 protocol, which distinguishes between a diagnosis of eMCI and lMCI, and includes rs-fMRI data. Study visits were approved by each site’s local Institutional Review Board. All participants provided informed consent. The following inclusion criteria were established by ADNI:

**MCI subjects:** subjective memory concern, clinical dementia rating (CDR) of 0.5, Mini-Mental State Exam (MMSE) score between 24 and 30, an abnormally low score on the Wechsler Memory Logical Memory II subscale (LM-II), no significant levels of impairment in other cognitive domains, preserved activities of daily living, non-demented.

*Early MCI***:** LM-II score between 9 and 11 for ≥16 years of education, 5–9 for 8–15 years of education, 3–6 for 0–7 years of education.

*Late MCI***:** LM-II score ≤ 8 for ≥16 years of education, ≤4 for 8–15 years of education, ≤2 for 0–7 years of education.

**CN subjects:** no subjective memory concern, CDR of 0, MMSE scores between 24 and 30, scores on LM-II within the expected range (≥9 for 16 or more years of education, ≥5 for 8–15 years of education, ≥3 for 0–7 years of education), no reported memory complaints, non-depressed, non-MCI, non-demented.

The distinction between early and late MCI was determined by ADNI via the extent of low performance on the LM-II subscale, such that an eMCI diagnosis was made for scores about 1 SD below the education adjusted norm, and a lMCI diagnosis for scores about 1.5 SD below the education adjusted norm, in addition to meeting the above MCI criteria^24^. In an independent sample, participants classified as eMCI displayed less severe cognitive impairment and a slower rate of progression than those classified as lMCI^3^. Participants were included in the current study if (1) they had a diagnosis of either CN, eMCI, or lMCI; (2) they were between 60 and 90 years old; (3) they had rs-fMRI and anatomical MRI collected on the same day; and (4) the images were collected using a 3 Tesla scanner. The first available scan that met these criteria was used for each subject. All participants that met these criteria were included in the study (*n* = 143).

### Image acquisition and preprocessing

Structural magnetization-prepared rapid gradient echo and rs-fMRI images were collected on a Philips Intera 3 Tesla scanner. Functional images were collected using a grandient echo pulse sequence (flip angle = 80°, slice thickness = 3.31 mm, echo time = 30 ms, repetition time = 3000 ms). Participants were instructed to keep their eyes open during resting-state scans.

Preprocessing steps were implemented in the MATLAB (R2017b) Conn toolbox (conn18b)^25^. Structural images underwent segmentation of gray matter, white matter, and cerebrospinal fluid. Functional images were preprocessed by realignment and unwarping, slice-timing correction, co-registration to structural images, spatial normalization, and motion outlier identification. White matter, cerebrospinal fluid, and 12 subject-motion parameters were included as nuisance regressors. Temporal band-pass filtering was employed to remove blood-oxygen-level dependent signal frequencies below 0.008 Hz or above 0.09 Hz. Outlier volumes were defined as having greater movement than 1.5 mm or a *Z* threshold of 7. Subjects with >50% of volumes removed were excluded from subsequent analyses (*n* = 13), resulting in a final sample of 130 subjects.

### Matrix construction and calculations of network measures

Functional time series were obtained using a functionally defined parcellation of 300 non-overlapping spherical regions of interest (ROIs)^26^, including substantial cortical, subcortical, and cerebellar coverage (Fig. 1C; coordinates available at https://wustl.app.box.com/s/twpyb1pflj6vrlxgh3rohyqanxbdpelw). Unweighted functional connectivity matrices were constructed for each subject with edges representing correlations between each ROI, by Fisher *Z* transformation and binarizing at thresholds selected for densities ranging from the top 2.5–25% of edges retained in each individual network.

#### Redundancy

A redundancy matrix (*R*) was calculated for each node pair from each subject’s connectivity matrix, defined as the sum of the direct and indirect edges between any two nodes (*i*, *j*), where *l* represents the total allowed path length and *L* represents the maximum path length (set to 4, in line with previous work^15^ and computational demands):

$$R\left( {i,j} \right) = \mathop {\sum}\limits_{l = 1}^L {p\left( {i,j,l} \right)} $$

The four hippocampal nodes of the parcellation were a priori defined as the main ROIs in this study: left and right anterior, along with the left and right posterior hippocampus. Redundancy was calculated between each hippocampal ROI and all other nodes (i.e., the average *R*-value for the 299 ROI-node pairs). Five additional regions were identified for secondary analysis, focused on nodes within two functional networks affected in early AD: the default mode and frontoparietal networks^27^ (see Supplementary Methods).

#### Degree

Unweighted degree was calculated for each hippocampal ROI, *i*, defined as the sum of all its binarized edges, where *d*_*i,j*_ represents the edge between nodes *i* and *j*.

$$k_i = \mathop {\sum }\limits_{j = 1}^n d_{i,j}$$

#### Global efficiency

Global efficiency, *E*_global_, was calculated from each subject’s binarized connectivity matrix, defined as the inverse of the shortest path length between two nodes (*i, j*), where *n* is the total number of nodes in the graph and *L*_*i,j*_ is the length of the shortest path between *i* and *j*:

$$E_{\mathrm{global}} = \frac{1}{{n(n - 1)}}\mathop {\sum }\limits_{i,j,j \ne i} \frac{1}{{L_{i,j}}}$$

### Cognition

We selected two cognitive domains that vary in the strength of their association with pathological aging: memory, the earliest reported cognitive deficit in MCI, and executive function, in which deficits are observed in later disease stages^28^. These were represented by composite measures MEM and EF, respectively^29,30^ (see Supplementary Methods).

### Participant characteristics

The final sample included 130 subjects in rs-fMRI analyses (39 CN, 54 eMCI, 37 lMCI; Fig. 1B). The groups did not differ in age, *F*(2,127) = 2.07, *p* = 0.130, or sex, *χ*^2^(2) = 4.04, *p* = 0.133. The groups differed in education, *F*(2,127) = 3.41, *p* = 0.036, such that the lMCI group had more years of education than the eMCI group, *p* = 0.036 (CN-eMCI *p* = 0.202, CN-lMCI: *p* = 0.734). Of this sample, 118 (37 CN, 50 eMCI, and 31 lMCI) had cognitive data within 3 months of their scan date and were included in the cognition analyses. Within this subset, the groups did not differ in age, *F*(2,115) = 2.66, *p* = 0.074, education, *F*(2, 115) = 2.47, *p* = 0.089, or sex, *χ*^2^(2) = 1.52, *p* = 0.468. The early and late MCI groups did not differ in percentage of amyloid-positive subjects, *χ*^2^(1) = 0.16, *p* = 0.686, 95% confidence interval (95% CI) [−0.17, 0.31] (see Supplementary Methods).

### Statistical analysis

Analyses were performed at all matrix densities (2.5% to 25%) and on the values averaged across densities. For brevity, results are reported in-text and in figures using the average across densities; results for each individual density are reported in the Supplementary Materials. Statistical analyses were run using R and MATLAB using raw data. Data were normalized for visualization.

#### Group comparisons

Permutation tests were used for group comparisons of graph measures, as they do not make assumptions about the distribution of the data and are more robust to non-normality than are parametric tests. Group comparisons were tested using the aovperm function from the permuco R package, which conducts analysis of covariance (ANCOVA) using permutation testing^31^. Three-group omnibus tests were first computed using redundancy or degree as the dependent variable, group as the independent variable, and education as a covariate. Each test was run using 10,000 permutations and a significance level of *p* < 0.05. Significant tests were followed by post hoc tests using the permutation analysis of variance (ANOVA) for each pairwise group comparison with 10,000 permutations. Education was only included as covariate in eMCI–lMCI comparisons, as the other groups did not differ in years of education. Multiple comparisons in post hoc testing were corrected for using the Benjamini and Hochberg procedure to reduce false discovery rate using the p.adjust R function. Cohen’s *d* and 95% CIs were calculated using the effsize R function for pairwise comparisons^32^.

Group differences in overall functional connectivity, white matter hyperintensities, and cognition were analyzed separately using a one-way ANOVA implemented with the aov R function, with a significance level of *p* < 0.05. Significant omnibus tests were probed using Tukey’s post hoc honest significant difference test using the TukeyHSD R function, which adjusts the *p*-values for multiple comparison testing at a significance level of *p* < 0.05. Education was included as a covariate in overall functional connectivity analyses, and age and education were included as covariates in white matter hyperintensity analyses, due to group demographic differences in the respective samples.

#### Nodal ratios

To quantify the magnitude of differences in redundancy between each group, pairwise nodal ratios were computed by dividing the average redundancy, *R*, of node *i* in one group by the average *R* of *i* in a second group. Redundancy of node *i* is the sum of its row in the redundancy matrix. This value was averaged to create a group-mean nodal redundancy value for each of the groups, which was then used to compute the ratio. This was done for all 300 nodes for each pairwise group comparison. For example, the average redundancy of each node for the CN subjects was divided by the redundancy of each node for the eMCI subjects, resulting in a CN : eMCI ratio for each of the 300 atlas nodes, such that a ratio of 1 indicates equivalent redundancies and a ratio >1 indicates higher redundancy in CN than in eMCI. This process was repeated for CN : lMCI and eMCI : lMCI comparisons. To test the significance of the posterior hippocampal nodal ranks, left and right posterior hippocampal ratios were averaged to create one posterior hippocampal ratio for each group ratio set and compared to the average ratio of a null distribution of random node pairs in each set, excluding posterior hippocampus (total: 10,000 random node pairs).

#### Redundancy regressions

Linear regressions were implemented using the lm R function, with redundancy as the independent variable and either cognition or global efficiency as the dependent variable, first collapsing across group, followed by within-group regressions. MEM and EF were regressed separately on hippocampal redundancy. Global efficiency was regressed on hippocampal redundancy, with education included as a covariate in the full sample regression. Hippocampal redundancy was regressed on MMSE within the MCI subjects to provide an alternate measure of MCI progression. Standardized βs are reported for all regression output. Due to the non-normality of the redundancy data, analyses were repeated using robust regression using Huber weights with the rlm function from the MASS R package^33^, with a Wald’s test of significance using the f.robftest function from the sfsmisc R package^34^.

## Results

### Lower hippocampal redundancy in MCI

**Fig. 2:**
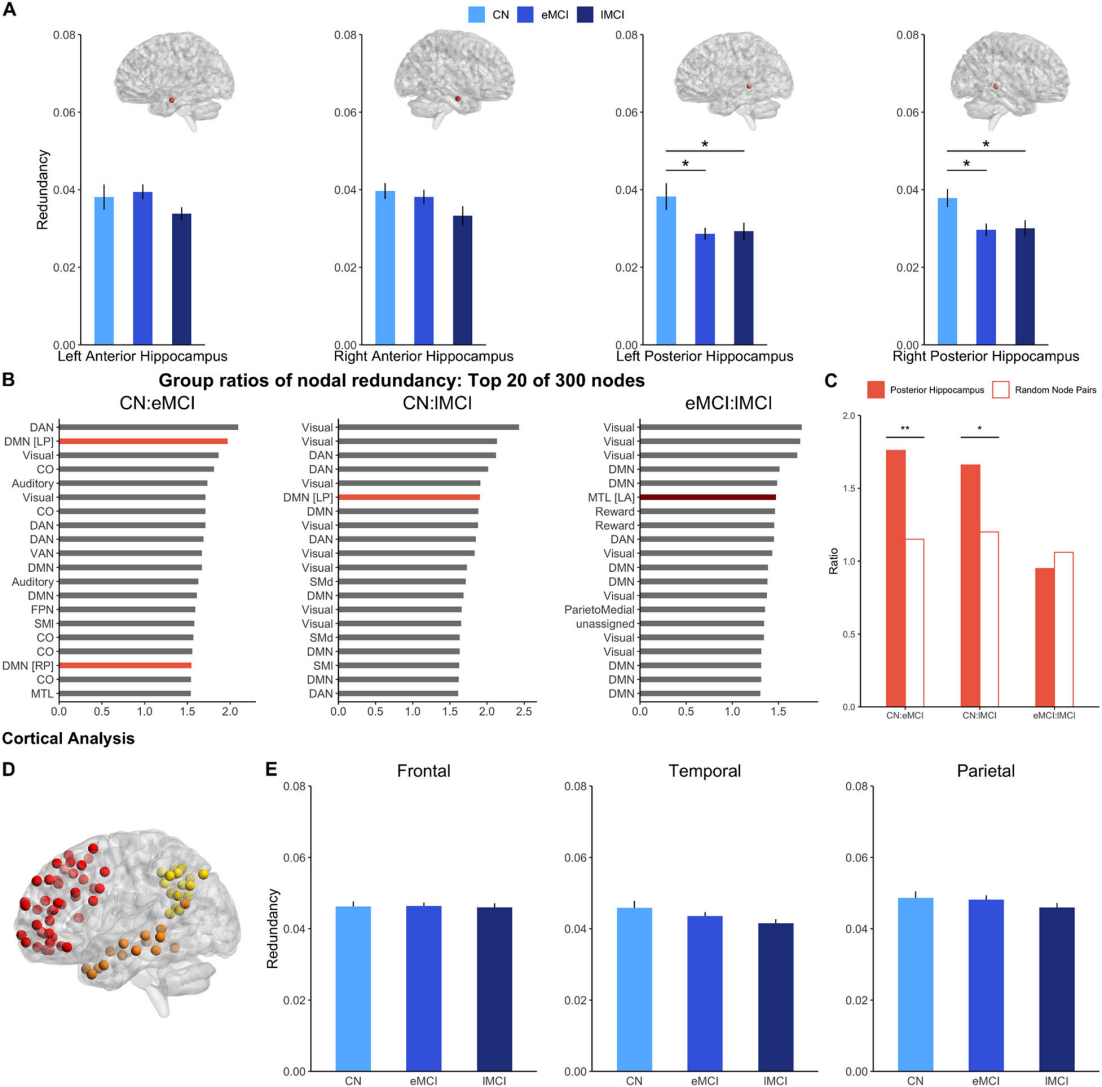
Hippocampal whole-brain functional redundancy differs in CN and MCI groups. **A** Group comparisons for the four hippocampal nodes. Error bars represent one SEM. **B** The top 20 of 300 nodal ratios in each ratio set. Posterior hippocampal nodes (peach) and anterior hippocampal nodes (dark red) are highlighted. Bar labels denote the node’s Seitzman et al.^26^ network affiliation. Hippocampal nodes are further labeled with “LP,” “RP,” “LA,” or “RA” to represent left posterior, right posterior, left anterior, and right anterior, respectively. **C** Statistical comparison of the average posterior hippocampal ratio to random node pairs in each ratio set. **D** Nodes included in the frontal (red), parietal (yellow), and temporal (orange) cortical analysis. **E** Group comparisons for the cortical nodes. Error bars represent one SEM. *p < 0.05, **p < 0.01.

We first compared hippocampal redundancy across healthy and pathological aging using an omnibus ANCOVA permutation test (Fig. 2A, Supplementary Fig. S1, and Supplementary Tables S1–3). Neither left nor right anterior hippocampal nodes significantly differed by group [left: *F*(2,126) = 1.43, *p* = 0.250; right: *F*(2, 126) = 2.15, *p* = 0.125]. Conversely, both posterior hippocampal nodes significantly differed by group [left: *F*(2,126) = 4.84, *p* = 0.009; right: *F*(2, 126) = 5.22, *p* = 0.004]. Post hoc tests revealed greater redundancy in the CN group as compared to either MCI group in both left [eMCI: *F*(1,91) = 7.76, *p* = 0.014, Cohen’s *d* = 0.59, 95% CI (0.16, 1.01); lMCI: *F*(1,74) = 4.67, *p* = 0.048, *d* = 0.50, 95% CI (0.03, 0.96)] and right [eMCI: *F*(1,91) = 9.00, *p* = 0.011, *d* = 0.63, 95% CI (0.20, 1.06); lMCI: *F*(1,74) = 6.21, *p* = 0.021, *d* = 0.57, 95% CI (0.11, 1.04)] posterior hippocampus. The MCI groups, on the other hand, did not differ in posterior hippocampal redundancy [left: *F*(1,88) = 0.09, *p* = 0.771, *d* = 0.05, 95% CI (−0.48, 0.37); right: *F*(1,88) = 0.02, *p* = 0.893, *d* = 0.03, 95% CI (−0.45, 0.39)].

To probe the relative importance of the observed group differences in hippocampal redundancy, we calculated between-group nodal redundancy ratio sets (CN : eMCI, CN : lMCI, eMCI : lMCI), thereby identifying the nodes with the greatest magnitude of group differences (top 20 presented in Fig. 2B). Posterior hippocampal nodes consistently appeared in the top 5% of nodes in the CN : eMCI and CN : lMCI sets across network densities (Supplementary Figs. S2–4). Left posterior hippocampus had the second highest ratio in the CN : eMCI set and the sixth highest ratio in the CN : lMCI set, indicating that not only does posterior hippocampal redundancy significantly differ between CN and MCI groups, but that posterior hippocampus has some of the largest differences across all nodes between CN and MCI groups. Indeed, posterior hippocampal ratios were significantly higher than the random node ratios in both the CN : eMCI set (*M*_posteriorHC_ = 1.76, *M*_random_ = 1.15, SE_random_ = 0.002, *p* = 0.001; Fig. 2C and Supplementary Table S4) and the CN : lMCI set (*M*_posteriorHC_ = 1.66, *M*_random_ = 1.20, SE_random_ = 0.002, *p* = 0.017). The posterior hippocampus ratio did not differ from the random nodes in the eMCI : lMCI set (*M*_posteriorHC_ = 0.95, *M*_random_ = 1.06, SE_random_ = 0.001, *p* = 0.831), supporting the finding that posterior hippocampal redundancy was less central for comparisons across MCI stages.

Although our analysis divided MCI into two groups based on LM-II scores, other ways exist to formalize MCI progression. Therefore, we additionally assessed the association between hippocampal redundancy and MMSE scores within the MCI participants. MMSE was not related to hippocampal redundancy in any of the four hippocampal nodes (lowest *p* = 0.204; Supplementary Fig. S5 and Supplementary Tables S5–6), consistent with the absence of group differences between early and lMCI.

To further validate the specific role of hippocampal redundancy in cognitive aging, we analyzed a set of frontal, temporal, and parietal nodes to determine whether the observed effects were widespread across cortical regions affected in early AD^27^ (Fig. 2D). Redundancy did not differ by group in any of the selected regions (lowest *p* = 0.097), suggesting this effect was, for the most part, unique to the hippocampus (Figs. 2E, Supplementary Fig. S6, and Supplementary Tables S7–8).

### Hippocampal redundancy is related to memory but not EF

**Fig. 3:**
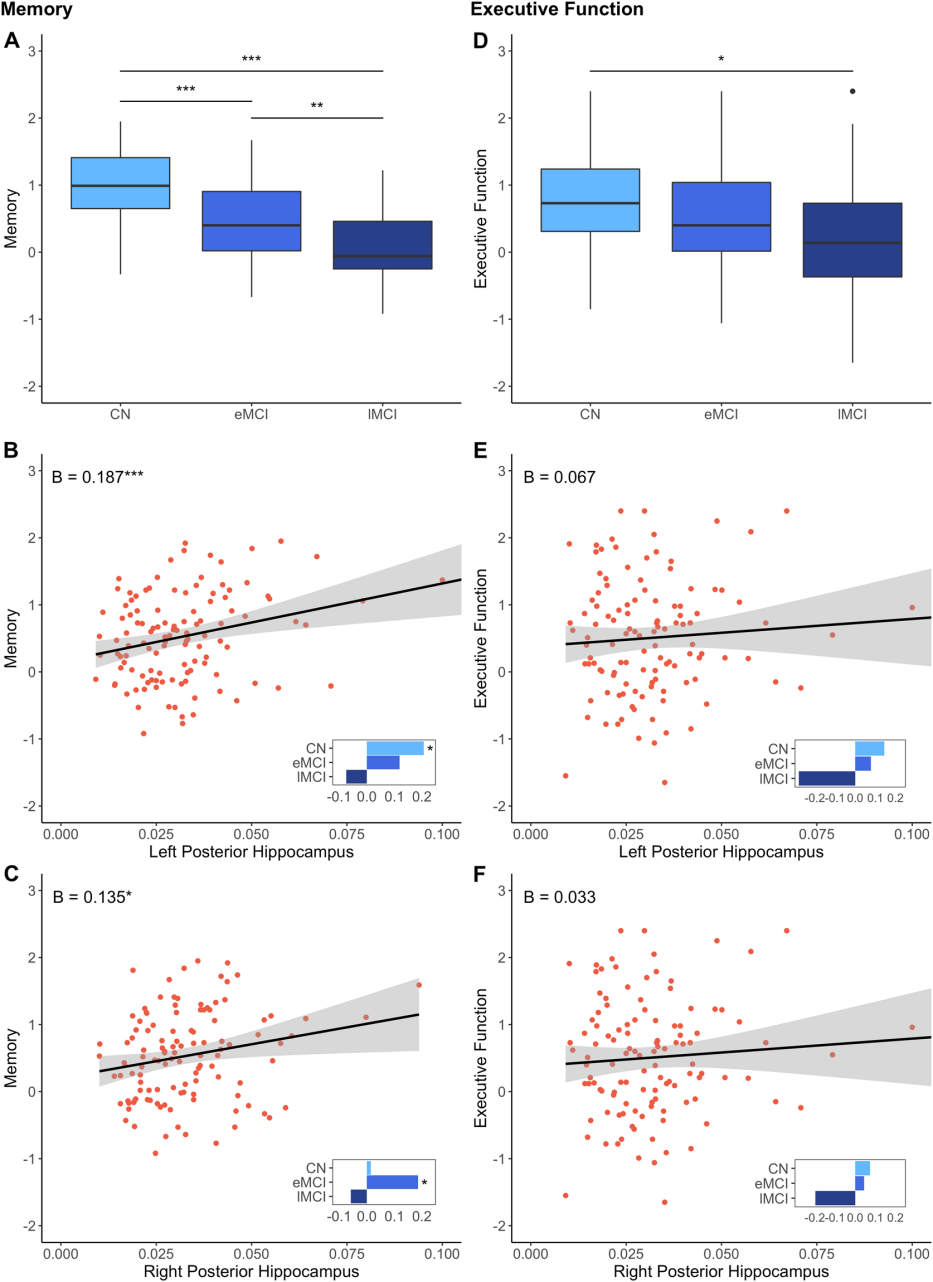
Relationship between redundancy and cognitive performance. **A** Memory composite scores across groups. Boxplot denotes the median (bold bar), first and third quartiles (box limits), and ±1.5 times the interquartile range (whiskers). **B** Whole sample regression of left posterior hippocampal redundancy on memory composite score. Inset shows within-group regression β-weights. **C** Whole sample regression of right posterior hippocampal redundancy on memory composite score. Inset shows within-group regression β-weights. **D** Executive function composite scores across cognitive groups. Boxplot denotes the median (bold bar), first and third quartiles (box limits), and ±1.5 times the interquartile range (whiskers). **E** Whole sample regression of left posterior hippocampal redundancy on executive function composite score. Inset shows within-group regression β-weights. **F** Whole sample regression of the right posterior hippocampal redundancy on executive function composite score. Inset shows within-group regression β-weights. *p < 0.05, **p < 0.01, ***p < 0.001.

We sought additional evidence of a protective role of redundancy in aging from the relationship between cognitive performance and redundancy, hypothesizing that functional redundancy would be associated with a cognitive benefit. The groups significantly differed in MEM scores [*F*(2, 115) = 24.60, *p* < 0.001, (Fig. 3A)], such that the CN group (*M* = 1.01, SD = 0.55) had higher scores than the eMCI group (*M* = 0.47, SD = 0.58, *p* < 0.001, 95% CI [−0.83, −0.25]) and the lMCI group (*M* = 0.07, SD = 0.53, *p* < 0.001, 95% CI [−1.26, −0.62]), and the eMCI group had higher scores than the lMCI group (*p* = 0.006, 95% CI [−0.70, −0.10]). We initially collapsed our sample into a single group to examine the overall relationship between cognition and redundancy, focusing on posterior hippocampal redundancy due to its consistency in group difference analyses and prominence in nodal rankings. Both left and right posterior hippocampal redundancy were related to higher MEM scores (Fig. 3B, C, Table 1, and Supplementary Figs. S9–10). Although the results reveal an overall effect of higher redundancy relating to better memory performance, we performed separate regressions by group as this relationship could differ based on cognitive status. The positive relationship between MEM and left hippocampal redundancy was retained in the CN group but not in either MCI group (Fig. 3B, C insets, Supplementary Fig. S7, Table 1, and Supplementary Table S9). In the right hippocampus, only the eMCI group showed a positive relationship, but significance was inconsistent across densities (Supplementary Fig. S7, Table 1, and Supplementary Table S10).

**Table 1:** Posterior hippocampal redundancy-cognition regressions for averaged density.

	Left posterior			Right Posterior		
	β	*p*-Value	Adjusted *R*^2^	β	*p*-Value	Adjusted *R*^2^
Memory						
Whole group	0.187	0.001	0.076	0.135	0.022	0.036
CN	0.206	0.023	0.115	0.014	0.877	0.028
eMCI	0.119	0.152	0.022	0.186	0.021	0.087
lMCI	−0.075	0.427	0.012	−0.058	0.530	0.020
Executive function						
Whole group	0.067	0.403	0.003	0.033	0.680	0.007
CN	0.143	0.274	0.007	0.073	0.571	0.019
eMCI	0.077	0.529	0.012	0.043	0.718	0.018
lMCI	−0.280	0.099	0.060	−0.198	0.237	0.015

This analysis process was repeated for EF. The groups differed in EF scores [*F*(2, 115) = 3.69, *p* = 0.028, (Fig. 3D)], such that the CN group (*M* = 0.78, SD = 0.77) had higher scores than the lMCI group (*M* = 0.22, SD = 0.97, *p* = 0.021, 95% CI [−1.06, −0.07]), but did not differ from the eMCI group (*M* = 0.49, SD = 0.84, *p* = 0.245, 95% CI [−0.74, 0.14]). The two MCI groups did not differ from each other (*p* = 0.370, 95% CI [−0.73, 0.20]). Posterior hippocampal redundancy was not related to EF scores in our full sample, nor in any group (Fig. 3E, F, Supplementary Fig. S7, Table 1, and Supplementary Tables S11–12). Results for both MEM and EF were consistent when using robust regression methods (Supplementary Table S13), suggesting the results were not driven by outliers.

### Specificity of redundancy as a topological property

**Fig. 4:**
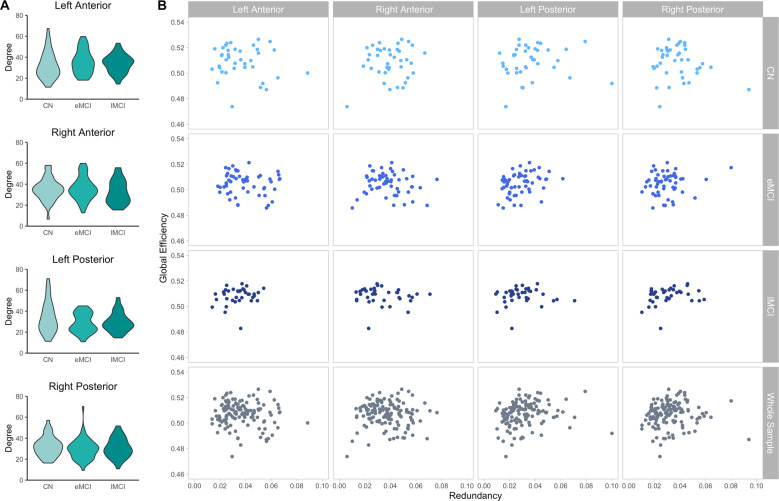
Degree and global efficiency. **A** Group comparisons of degree in each hippocampal ROI. **B** Global efficiency regressed on hippocampal redundancy in each ROI within each group and collapsing across groups.

As the redundancy measure includes paths of length-1 (i.e., direct connections), we next examined whether similar group differences would be observed using only these length-1 paths by way of node degrees (e.g., whether the inclusion of indirect paths is informative). No significant group differences were observed for any of the hippocampal nodes [left anterior: *F*(2, 126) = 0.94, *p* = 0.390; right anterior: *F*(2, 126) = 0.80, *p* = 0.455; left posterior: *F*(2, 126) = 2.74, *p* = 0.071; right posterior: *F*(2, 126) = 0.90, *p* = 0.413; Fig. 4A and Supplementary Table S14].

Groups were compared on overall functional connectivity for each of the hippocampal nodes to ensure graph measures were not biased due to underlying functional connectivity differences^35^ (see Supplementary Methods). No group differences were found when retaining only positive correlations [left anterior: *F*(2, 126) = 1.15, *p* = 0.319; right anterior: *F*(2, 126) = 1.68, *p* = 0.191; left posterior: *F*(2, 126) = 2.58, *p* = 0.080; right posterior: *F*(2, 126) = 1.31, *p* = 0.274], or when taking the absolute value of all correlations, [left anterior: *F*(2, 126) = 0.55, *p* = 0.580; right anterior: *F*(2, 126) = 0.72, *p* = 0.489; left posterior: *F*(2, 126) = 0.39, *p* = 0.679; right posterior: *F*(2, 126) = 0.05, *p* = 0.954].

In addition, there were no group differences in total volume of white matter hyperintensities, *F*(2, 109) = 0.31, *p* = 0.737, nor did volume of white matter hyperintensities relate to hippocampal redundancy in any of the four ROIs (Supplementary Table S15). Together, these results suggest that the redundancy measure provides valuable and specific information differentiating CN and MCI individuals.

### Redundancy does not come at the cost of efficiency

Our final analysis examined whether the existence of redundant edges in brain networks is associated with compromised communication efficiency within the network, measured by global efficiency. There was no significant relationship between global efficiency and redundancy in any of the hippocampal nodes when collapsing across group [left anterior: *β* = −0.17, *p* = 0.061, adjusted *R*^2^ = 0.02; right anterior: *β* = −0.03, *p* = 0.766, adjusted *R*^2^ = 0.01; left posterior: *β* = 0.07, *p* = 0.411, adjusted *R*^2^ = 0.003; right posterior: *β* = 0.05, *p* = 0.573, adjusted *R*^2^ = 0.01] (Fig. 4B and Supplementary Table S16), suggesting that efficient network communication is not compromised by having high functional redundancy. We further probed redundancy-efficiency relationships within each group, finding no significant relationships in either the CN or lMCI groups (Fig. 4B and Supplementary Tables S17–S19). There was a positive relationship between global efficiency and left posterior hippocampal redundancy in the eMCI group, *β* = 0.32, *p* = 0.017, adjusted *R*^2^ = 0.09. Results were consistent using robust regression methods (Supplementary Table S20).

## Discussion

Certain individuals exhibit normal cognition despite harboring the characteristic pathology of AD and other dementias^5,36,37^, yet mechanisms of neuroprotection in the human brain remain elusive and difficult to quantify. Early work postulated that redundancy may exist in the brain, but it could not be quantified with the contemporary methods^12^. In the current study, we quantified functional redundancy in healthy older adults and those with either early- or late-stage MCI to test whether redundancy acts as a neuroprotective mechanism against pathological aging. Consistent with previous reports of beneficial redundancy in biological systems^13,14^, we found evidence that redundancy serves a neuroprotective role in cognitive aging. Specifically, healthy older adults showed higher posterior hippocampal redundancy than individuals with MCI, and posterior hippocampal redundancy was positively related to memory performance, with this association primarily driven by the cognitively intact group. The MCI groups did not differ in levels of hippocampal redundancy nor did they exhibit relationships between redundancy and cognition, thereby supporting the conclusion that redundancy incurs a neuroprotective benefit in healthy (and possibly asymptomatic) aging, which plateaus in symptomatic pathological aging (Fig. 1D, red line). Further, we found no group differences in temporal, parietal, or frontal cortical nodes, suggesting these results are mostly specific to the hippocampus.

We found a clear distinction between anterior and posterior hippocampus, such that only posterior hippocampus consistently differentiated healthy aging from MCI. This distinction may be explained by the functional specialization along the long-axis of the hippocampus. As established in the rodent literature, dorsal (posterior in primates) hippocampus underlies memory processes, and ventral (anterior in primates) hippocampus is involved in emotional processing. The anatomical connectivity of these regions supports this functional segregation, with ventral hippocampus connecting to the amygdala and dorsal hippocampus connecting to retrosplenial and anterior cingulate cortices^38^. This distinction is further supported by a prominent memory theory^39^, differentiating between a posterior medial and an anterior temporal functional network in humans, which largely overlap with the previously described structural connections^38^. Relatedly, posterior, but not anterior, hippocampal nodes clustered with the default mode network (DMN) in the parcellation employed in the current study^26^. The DMN has considerable overlap with the proposed posterior medial network^39^ and exhibits functional deficits in the context of aging and AD^40–42^, thereby supporting our primary findings in posterior rather than anterior hippocampus.

In addition to group differences in redundancy, we found that posterior hippocampal redundancy is related to memory performance. This relationship held in both our full sample and in healthy older adults but not in either MCI group. It is possible that the MCI groups, as they on average have lower redundancy than the CN group, may not exhibit enough hippocampal redundancy to benefit performance. Another interpretation is that the relationship between redundancy and cognition differs across groups. Although neither reached significance, the eMCI group had a positive memory-redundancy relationship, unlike the lMCI group that exhibited a negative relationship. Future work should probe whether there is an amount of redundancy that is necessary to benefit cognition or if redundancy rather becomes a hindrance in later disease stages. We did not find any relationship between hippocampal redundancy and EF. Although hippocampal function has been associated with a range of cognitive processes^43^, its role is particularly critical to mnemonic processes^44,45^. This dissociation, then, indicates a selective cognitive benefit of nodal functional redundancy, which can be further explored in other brain regions (e.g., prefrontal cortex and EF).

Taken together, these results provide support for redundancy as a quantifiable neuroprotective mechanism, but further research is needed to satisfactorily describe its role as either a reserve or compensatory mechanism^6,7^. Reserve encompasses both structural and functional properties of the brain accumulated over time that support cognitive or clinical function in the event of damage, as opposed to a compensatory mechanism that reacts in response to damage^6–8^. We would expect increased redundancy in MCI if it acted as compensatory response; rather, we observed a benefit in healthy aging, suggesting it serves as a reserve mechanism. AD-type pathology is already present in healthy aging and MCI stages, particularly in the hippocampus^5^; aging individuals with more reserve (e.g., redundancy) are likely to show better cognitive outcomes, and therefore exhibit resilience to that damage. However, it is possible that redundancy increases for some individuals in healthy aging as a compensatory response to the accumulation of early pathology, which does not occur in individuals who are subsequently diagnosed with MCI or AD. Lifespan or longitudinal studies can provide additional evidence to elucidate its exact role.

Several limitations exist in this study. Although our results suggest a cognitive benefit of functional redundancy, our data were cross-sectional, limiting our claims about the progression of redundancy during the course of aging. Future studies should investigate longitudinally and probe the potential difference in anterior and posterior hippocampal redundancy in differentiating stages of healthy and pathological aging, as later stages of MCI and AD may be expected to differ from healthy controls in anterior-based mnemonic processes^46,47^. In fact, our results demonstrated anterior hippocampal nodes are consistently among the highest 5% of nodal ratios between MCI groups. However, we did not observe significant group differences. The definition of early and late MCI used here, adopted from the ADNI protocol, may have precluded our ability to observe differences between MCI stages. The early versus late distinction is determined solely on performance on the LM-II, which could be affected by factors other than later stage cognitive decline (e.g., fatigue, concentration, practice effects)^48,49^. However, we have no reason to believe these factors would differentially affect the groups, and we did not find a relationship between hippocampal redundancy and MMSE scores in MCI. Recently, it was proposed that an accurate staging of MCI and AD progression can be achieved through a combination of amyloid-β, tau, and neurodegenerative markers^50^. The shift from staging MCI based on symptomatic markers to biological markers should be considered in future investigations.

In conclusion, we found that posterior hippocampal redundancy is greater in healthy or asymptomatic older adults than in individuals with MCI. Our data suggest a decrease in redundancy between healthy aging and MCI, upon which the amount of redundancy plateaus and no longer provides a functional advantage. Further, higher amounts of hippocampal redundancy are related to better memory performance. Although previous discussions of redundancy have been wary of a trade-off with efficiency^12^, we did not observe any reduction in efficiency as a result of hippocampal redundancy. These data provide novel and promising quantitative support that redundancy acts as a neuroprotective mechanism in cognitive aging.

## Supplementary information

Langella SUPPLEMENTAL MATERIAL

## Data Availability

Neuroimaging and cognitive data are available at http://adni.loni.usc.edu/. The datasets generated in the current study are available from the corresponding author upon reasonable request. Data used in preparation of this article were obtained from the Alzheimer’s Disease Neuroimaging Initiative (ADNI) database (adni.loni.usc.edu). As such, the investigators within the ADNI contributed to the design and implementation of ADNI and/or provided data but did not participate in analysis or writing of this report. A complete listing of ADNI investigators can be found at http://adni.loni.usc.edu/wp-content/uploads/how_to_apply/ADNI_Acknowledgement_List.pdf.
