# Supplementary material for: Lower functional hippocampal redundancy in mild cognitive impairment
Source: Transl Psychiatry. 2021 Jan 18;11:61. doi: 10.1038/s41398-020-01166-w (PMC7813821; doi:10.1038/s41398-020-01166-w)
Supplement: Supplementary file 1 — Langella SUPPLEMENTAL MATERIAL [file 41398_2020_1166_MOESM1_ESM.docx]

Supplementary Materials for:

**Lower functional hippocampal redundancy in mild cognitive impairment**

Stephanie Langella, M.A, Muhammad Usman Sadiq, Ph.D., Peter J. Mucha, Ph.D., Kelly S. Giovanello, Ph.D., and Eran Dayan, Ph.D. for the Alzheimer’s Disease Neuroimaging Initiative

**Supplementary Methods**

**Overall functional connectivity**

Overall levels of functional connectivity were calculated for each of the four hippocampal nodes for each subject using (1) all positive correlations and (2) the absolute value of all network correlations, as underlying differences in connectivity may bias patient group comparisons when using proportional thresholding.

**Total volume of white matter hyperintensities**

Total volume of white matter hyperintensities were available for 114 subjects through ADNI. Volumes were calculated using a Bayesian approach to segment the T1 and fluid attenuation inversion recovery (FLAIR) MR scan sequences (additional protocol information available through ADNI).

**Classification of Amyloid-Beta positivity**

Florbetapir PET imaging was available for 81 of the 91 MCI subjects within 1 year of their resting-state scan. Mean florbetapir uptake was calculated for cortical gray matter and normalized using a cerebellar reference region. Subjects with normalized florbetapir uptake equal to or above 1.11 were classified as amyloid-positive, and those below 1.11 were classified as amyloid-negative^1,2^ (additional protocol information available through ADNI). Of the 81 MCI participants with available amyloid-beta PET imaging, 50 were amyloid-positive and 31 were amyloid-negative.

**Secondary nodal analysis**

We performed a secondary analysis on precuneus, anterior cingulate cortex (ACC), frontal, temporal, and parietal cortical nodes. Analyzed nodes were clustered with either the default mode network or frontoparietal network. Precuneus consisted of six nodes (three from each hemisphere), and ACC was comprised of four nodes (three left hemisphere, one right hemisphere). Both precuneus and ACC were solely comprised of nodes clustering with the default mode network. Frontal (*n* = 44), temporal (*n* = 15), and parietal (*n* = 18) nodes were clustered with default mode and frontoparietal networks. Redundancy was calculated separately for each node, then averaged across all nodes comprising the anatomical region.

**Cognitive Composite Scores**

MEM and EF were calculated using an IRT framework^3^. MEM incorporated RAVLT (Trials 1-5, Interference, Immediate recall, Delay, Recognition), ADAS-Cog (Trials 1-3, Recall, Recognition), Logical Memory (Immediate, Delay), MMSE (word recall). EF was calculated using: Category Fluency (animals, vegetables), WAIS-R Digit Symbol, Digit Span Backwards, Trails A, Trails B, Clock Drawing. Both MEM and EF have a mean of 0 and standard deviation of 1, with positive scores indicating better performance.

**Supplementary Figures**

Figure S1: Whole-brain hippocampal redundancy across densities. **p* < .05, ***p* < .01. CN-eMCI comparison in black, CN-lMCI comparison in red.


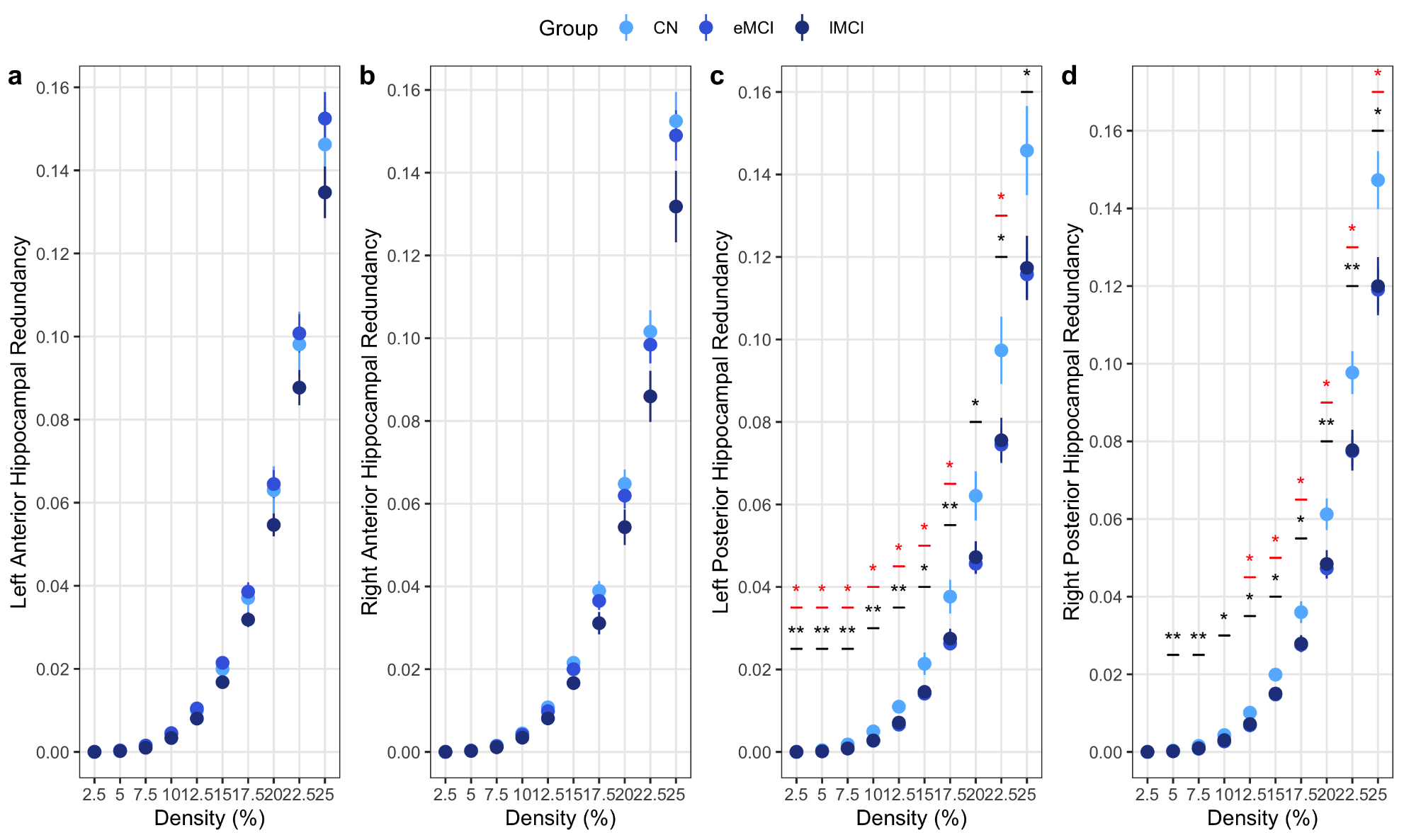


Figure S2: CN:eMCI nodal ratios across densities. Posterior hippocampal nodes in peach.


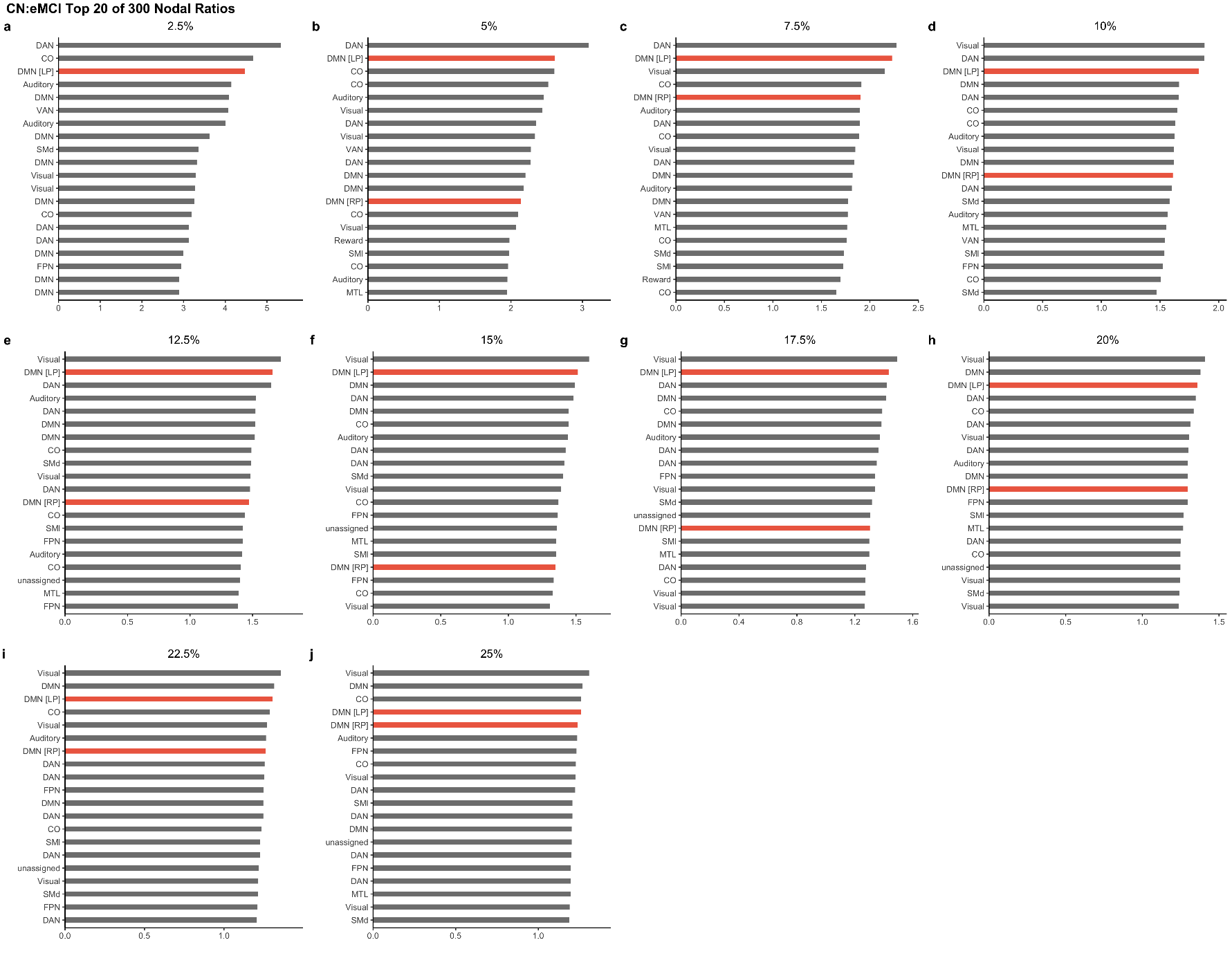


Figure S3: CN:lMCI nodal ratios across densities. Posterior hippocampal nodes in peach, anterior hippocampal nodes in dark red.


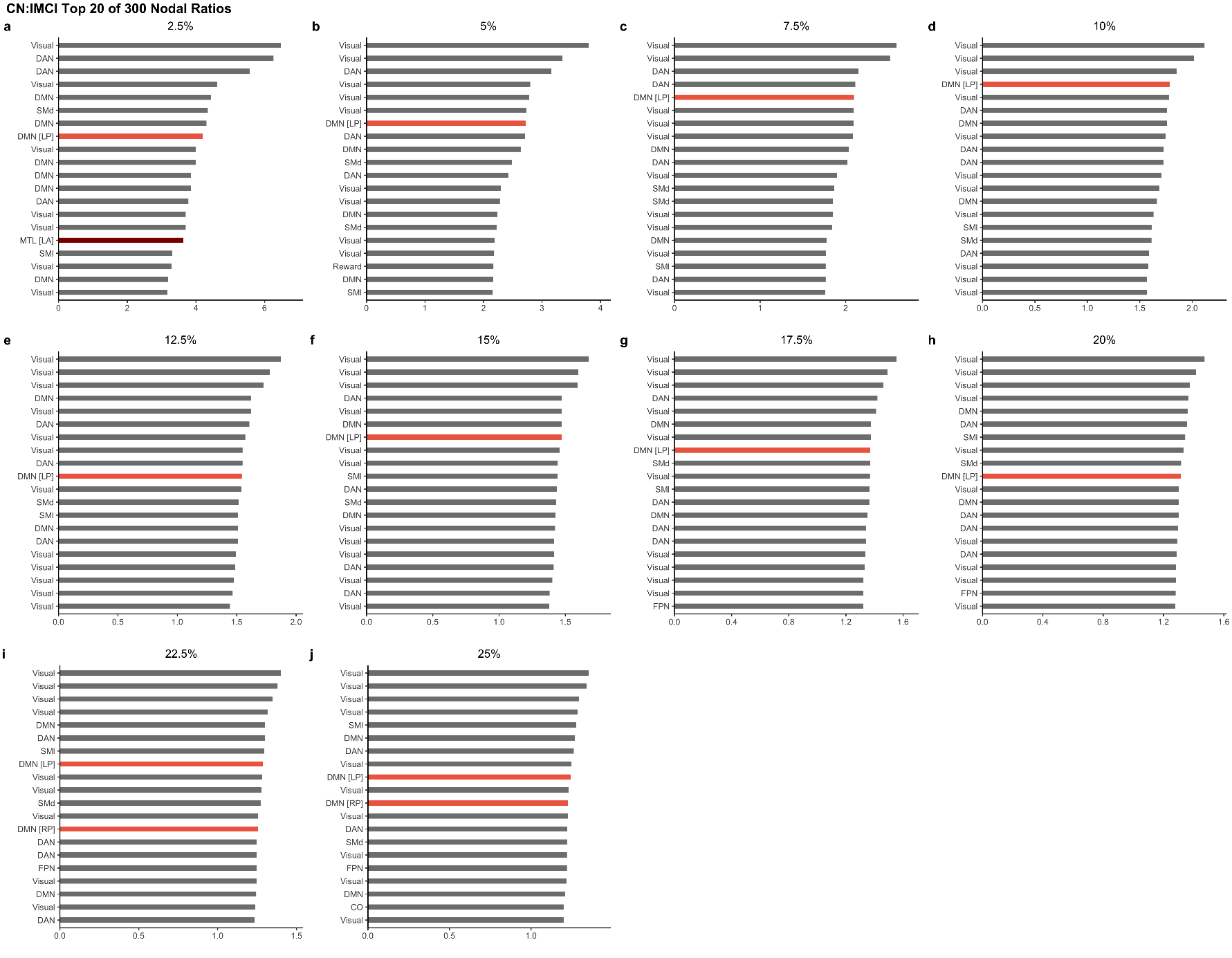


Figure S4: eMCI:lMCI nodal ratios across densities. Anterior hippocampal nodes in dark red.


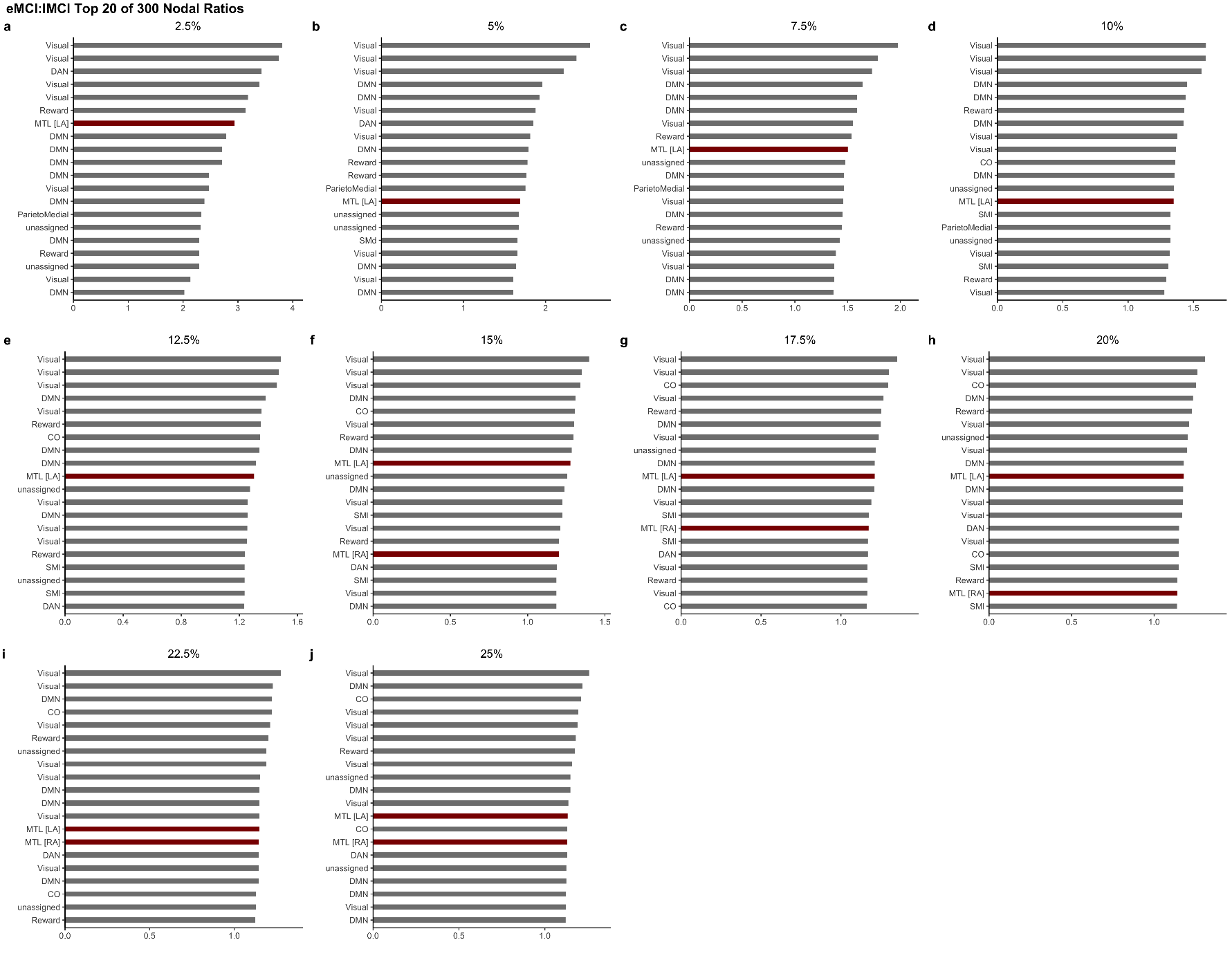


Figure S5: Scatterplots showing hippocampal redundancy-MMSE relationship in MCI subjects


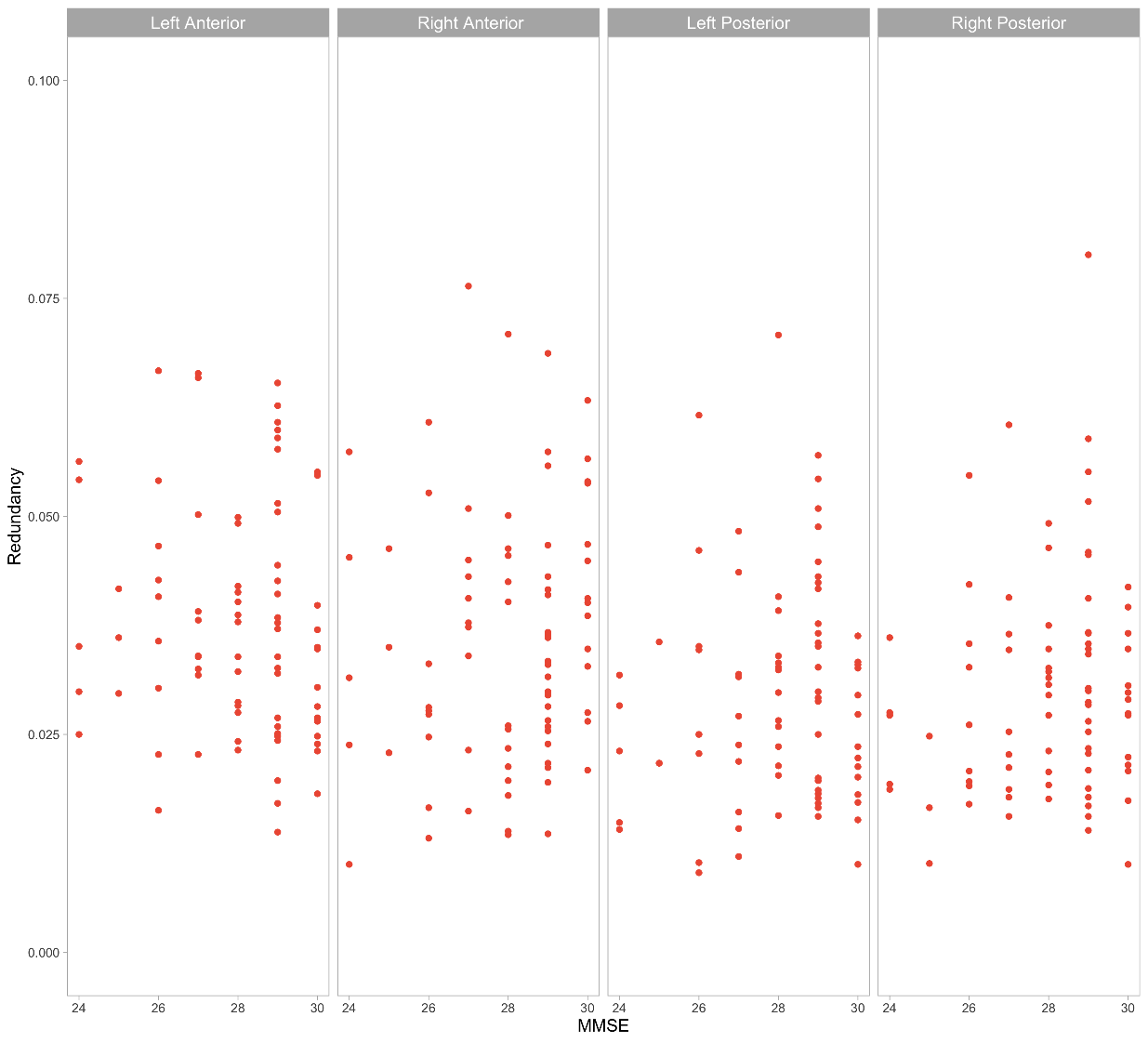


Figure S6: Precuneus and anterior cingulate cortex (ACC) redundancy group difference comparisons. (A) Precuneus nodes in yellow, ACC nodes in red. (B) Group means with standard error bars representing one standard error of the mean.


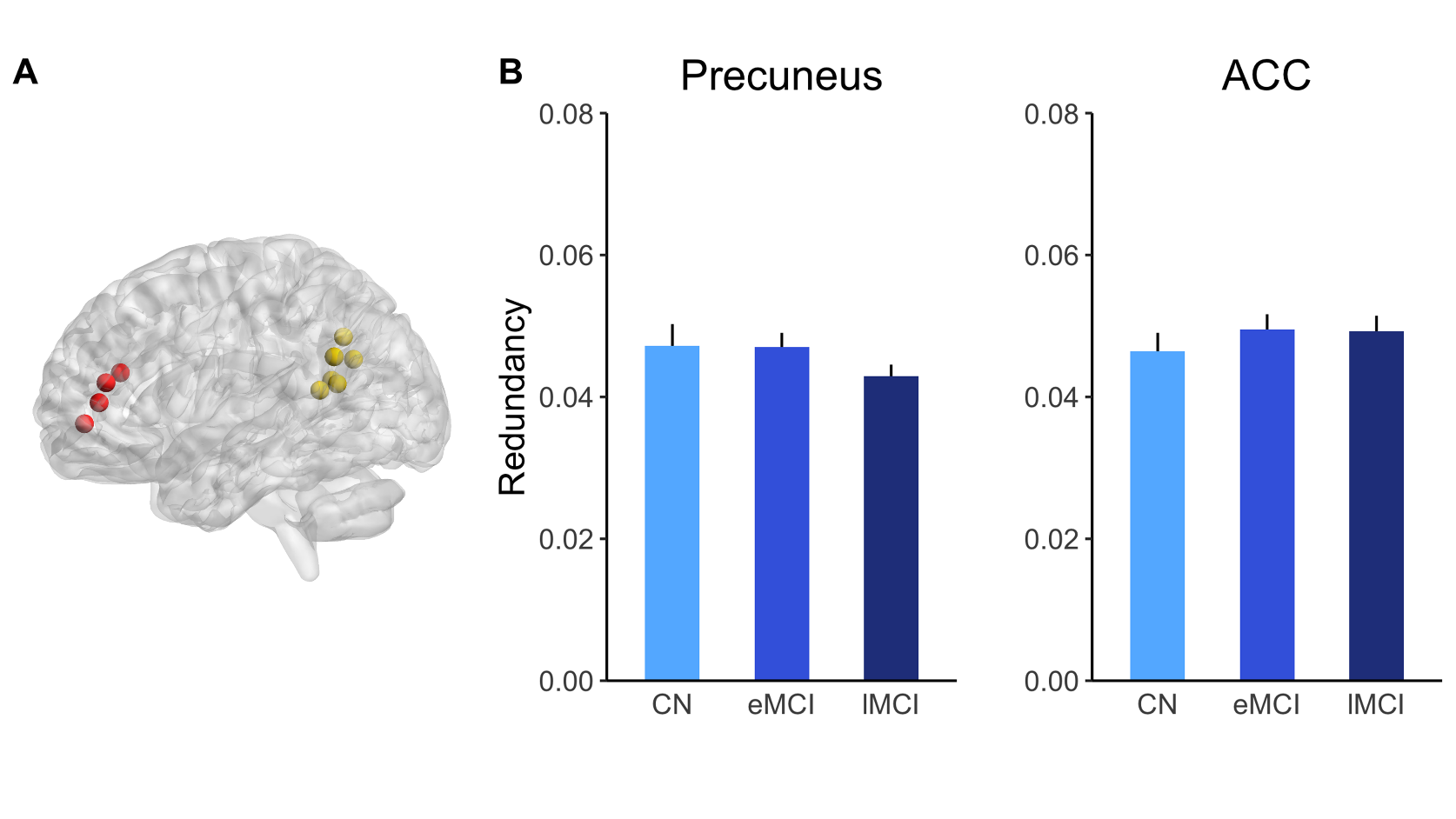


Figure S7: Within-group scatterplots of hippocampal redundancy-cognition relationships.


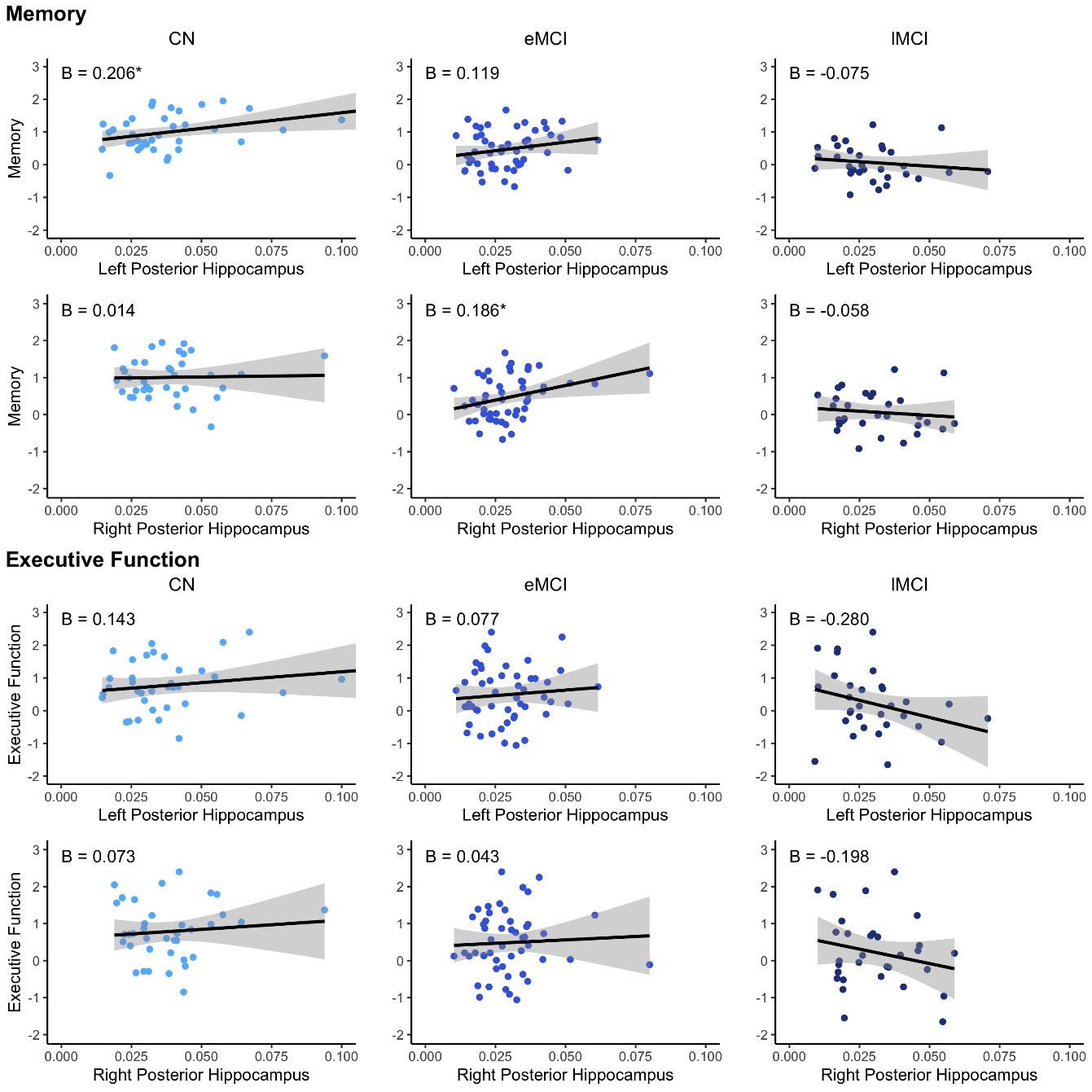


**Supplementary Tables**

Table S1

*Hippocampal redundancy omnibus test statistics across densities (df = 2, 126)*

|  | Left Anterior | | Right Anterior | | Left Posterior | | Right Posterior | |
| --- | --- | --- | --- | --- | --- | --- | --- | --- |
| Density | *F* | *p* | *F* | *p* | *F* | *p* | *F* | *p* |
| 2.5 | 1.02 | .373 | 1.82 | .162 | 4.39 | .002 | 2.15 | .119 |
| 5 | 1.13 | .337 | 1.43 | .254 | 6.73 | .002 | 4.49 | .008 |
| 7.5 | 1.43 | .252 | 1.03 | .358 | 6.22 | .001 | 5.33 | .005 |
| 10 | 1.29 | .285 | 1.33 | .270 | 5.66 | .003 | 4.73 | .008 |
| 12.5 | 1.45 | .239 | 2.07 | .130 | 5.37 | .004 | 4.89 | .007 |
| 15 | 1.72 | .186 | 2.35 | .098 | 5.36 | .004 | 4.12 | .018 |
| 17.5 | 1.50 | .225 | 2.40 | .094 | 5.12 | .005 | 4.20 | .015 |
| 20 | 1.49 | .232 | 1.93 | .152 | 4.78 | .008 | 5.00 | .008 |
| 22.5 | 1.32 | .277 | 2.10 | .126 | 4.70 | .010 | 5.50 | .005 |
| 25 | 1.29 | .279 | 2.04 | .130 | 4.27 | .014 | 5.33 | .006 |

Table S2

*Left posterior hippocampal redundancy pairwise comparison’s test statistics across densities with FDR adjusted p values*

|  | CN-eMCI (df = 1, 91) | | | CN-lMCI (df = 1, 74) | | | eMCI-lMCI (df = 1, 88) | | |
| --- | --- | --- | --- | --- | --- | --- | --- | --- | --- |
| Density | *F* | *p* | Adjusted *p* | *F* | *p* | Adjusted *p* | *F* | *p* | Adjusted *p* |
| 2.5 | 5.40 | .003 | .008 | 3.57 | .028 | .042 | 0.02 | .894 | .894 |
| 5 | 8.25 | .003 | .008 | 6.32 | .008 | .012 | 0.04 | .839 | .839 |
| 7.5 | 8.56 | .001 | .004 | 5.30 | .017 | .025 | 0.04 | .841 | .841 |
| 10 | 7.93 | .002 | .007 | 5.14 | .022 | .032 | 0.00 | .960 | .960 |
| 12.5 | 8.24 | .003 | .009 | 4.40 | .033 | .050 | 0.27 | .604 | .604 |
| 15 | 8.02 | .004 | .011 | 4.90 | .027 | .040 | 0.09 | .771 | .771 |
| 17.5 | 8.29 | .003 | .008 | 4.53 | .032 | .048 | 0.21 | .648 | .648 |
| 20 | 7.94 | .006 | .017 | 4.30 | .040 | .060 | 0.18 | .673 | .673 |
| 22.5 | 7.52 | .006 | .018 | 4.79 | .032 | .048 | 0.05 | .827 | .827 |
| 25 | 6.99 | .009 | .026 | 4.47 | .036 | .054 | 0.05 | .826 | .826 |

Table S3

*Right posterior hippocampal redundancy pairwise comparison’s test statistics across densities with FDR adjusted p values*

|  | CN-eMCI (df = 1, 91) | | | CN-lMCI (df = 1, 74) | | | eMCI-lMCI (df = 1, 88) | | |
| --- | --- | --- | --- | --- | --- | --- | --- | --- | --- |
| Density | *F* | *p* | Adjusted *p* | *F* | *p* | Adjusted *p* | *F* | *p* | Adjusted *p* |
| 5 | 6.15 | .003 | .010 | 3.12 | .063 | .095 | 0.55 | .466 | .466 |
| 7.5 | 8.11 | .002 | .006 | 3.84 | .043 | .065 | 0.68 | .405 | .405 |
| 10 | 7.69 | .005 | .014 | 3.78 | .054 | .081 | 0.54 | .469 | .469 |
| 12.5 | 7.76 | .005 | .015 | 5.04 | .027 | .041 | 0.11 | .741 | .741 |
| 15 | 6.49 | .010 | .030 | 4.85 | .026 | .039 | 0.02 | .885 | .885 |
| 17.5 | 6.78 | .010 | .031 | 5.08 | .023 | .035 | 0.01 | .910 | .910 |
| 20 | 8.85 | .002 | .007 | 5.52 | .024 | .036 | 0.07 | .792 | .792 |
| 22.5 | 9.54 | .002 | .006 | 6.82 | .011 | .016 | 0.02 | .961 | .961 |
| 25 | 9.48 | .003 | .008 | 6.64 | .011 | .016 | 0.01 | .935 | .935 |

Table S4

*Mean group nodal ratios for posterior hippocampus (HC) and random node (RN) pairs with associated standard error of the mean (SEM) and p-value*

|  | CN:eMCI | | | | CN:lMCI | | | | eMCI:lMCI | | | |
| --- | --- | --- | --- | --- | --- | --- | --- | --- | --- | --- | --- | --- |
| Density | HC mean | RN mean | RN SEM | *p* | HC mean | RN mean | RN SEM | *p* | HC mean | RN mean | RN SEM | *p* |
| 2.5 | 3.19 | 1.57 | .005 | .012 | 2.89 | 1.74 | .006 | .050 | 0.89 | 1.21 | .004 | .824 |
| 5 | 2.38 | 1.28 | .003 | .001 | 2.27 | 1.38 | .003 | .018 | 0.95 | 1.11 | .002 | .793 |
| 7.5 | 2.06 | 1.17 | .002 | < .001 | 1.86 | 1.24 | .002 | .012 | 0.90 | 1.08 | .001 | .907 |
| 10 | 1.72 | 1.12 | .002 | .001 | 1.61 | 1.16 | .002 | .011 | 0.93 | 1.05 | .001 | .861 |
| 12.5 | 1.57 | 1.09 | .001 | < .001 | 1.47 | 1.13 | .001 | .014 | 0.94 | 1.04 | .001 | .850 |
| 15 | 1.43 | 1.07 | .001 | .001 | 1.40 | 1.10 | .001 | .009 | 0.98 | 1.03 | .001 | .748 |
| 17.5 | 1.37 | 1.06 | .001 | .001 | 1.33 | 1.08 | .001 | .011 | 0.97 | 1.02 | .001 | .777 |
| 20 | 1.33 | 1.05 | .001 | .001 | 1.29 | 1.06 | .001 | .007 | 0.97 | 1.02 | .001 | .793 |
| 22.5 | 1.28 | 1.04 | .001 | < .001 | 1.27 | 1.05 | .001 | .004 | 0.99 | 1.01 | .001 | .682 |
| 25 | 1.25 | 1.03 | .001 | < .001 | 1.24 | 1.04 | .001 | .003 | 0.99 | 1.01 | .000 | .697 |

Table S5

*Hippocampal redundancy-MMSE regressions across densities*

|  | Left Anterior | | Right Anterior | | Left Posterior | | Right Posterior | |
| --- | --- | --- | --- | --- | --- | --- | --- | --- |
| Density | ß | *p* | ß | *p* | ß | *p* | ß | *p* |
| Avg. | -0.12 | .299 | 0.11 | .341 | 0.06 | .567 | 0.14 | .204 |
| 2.5 | -0.06 | .567 | 0.09 | .390 | 0.03 | .805 | 0.05 | .622 |
| 5 | -0.03 | .804 | 0.10 | .358 | 0.04 | .715 | 0.07 | .502 |
| 7.5 | -0.02 | .849 | 0.08 | .465 | 0.09 | .435 | 0.05 | .675 |
| 10 | -0.04 | .741 | 0.11 | .325 | 0.09 | .440 | 0.05 | .615 |
| 12.5 | -0.03 | .785 | 0.12 | .294 | 0.07 | .555 | 0.07 | .511 |
| 15 | -0.07 | .521 | 0.11 | .319 | 0.07 | .527 | 0.10 | .356 |
| 17.5 | -0.09 | .396 | 0.11 | .335 | 0.06 | .601 | 0.11 | .293 |
| 20 | -0.14 | .204 | 0.12 | .285 | 0.08 | .487 | 0.13 | .236 |
| 22.5 | -0.13 | .219 | 0.09 | .423 | 0.06 | .559 | 0.15 | .177 |
| 25 | -0.12 | .296 | 0.10 | .355 | 0.05 | .632 | 0.16 | .142 |

Table S6

*Hippocampal redundancy-MMSE regressions for averaged density, using robust regression (Huber weighting) and Wald test for significance*

|  | ß | *t* | *p* |
| --- | --- | --- | --- |
| Left Anterior | -0.13 | 1.13 | .258 |
| Right Anterior | 0.12 | 1.06 | .294 |
| Left Posterior | 0.07 | 0.66 | .511 |
| Right Posterior | 0.13 | 1.32 | .186 |

Table S7

*Cortical redundancy omnibus test statistics across densities (df = 2, 126)*

|  | Precuneus | | ACC | | Frontal | | Temporal | | Parietal | |
| --- | --- | --- | --- | --- | --- | --- | --- | --- | --- | --- |
| Density | *F* | *p* | *F* | *p* | *F* | *p* | *F* | *p* | *F* | *p* |
| Avg. | 1.12 | .337 | 0.78 | .475 | 0.20 | .821 | 2.34 | .097 | 0.94 | .399 |
| 2.5 | 1.85 | .160 | 0.86 | .446 | 0.42 | .670 | 4.08 | .002 | 0.81 | .457 |
| 5 | 1.95 | .146 | 1.06 | .354 | 0.45 | .642 | 4.81 | .005 | 0.85 | .434 |
| 7.5 | 1.76 | .180 | 1.13 | .329 | 0.33 | .722 | 4.16 | .011 | 0.99 | .376 |
| 10 | 1.53 | .219 | 1.03 | .360 | 0.29 | .771 | 3.69 | .022 | 0.93 | .400 |
| 12.5 | 1.35 | .272 | 1.00 | .364 | 0.25 | .787 | 3.19 | .039 | 0.83 | .449 |
| 15 | 1.36 | .260 | 0.79 | .462 | 0.21 | .829 | 2.86 | .054 | 0.83 | .447 |
| 17.5 | 1.24 | .293 | 0.77 | .479 | 0.19 | .841 | 2.66 | .074 | 0.92 | .405 |
| 20 | 1.11 | .335 | 0.83 | .440 | 0.18 | .840 | 2.31 | .104 | 0.99 | .379 |
| 22.5 | 1.03 | .367 | 0.70 | .499 | 0.18 | .837 | 2.12 | .124 | 0.97 | .378 |
| 25 | 0.90 | .413 | 0.73 | .489 | 0.23 | .801 | 2.06 | .127 | 0.88 | .421 |

Table S8

*Temporal node redundancy pairwise comparison’s test statistics across densities (with significant omnibus) with FDR adjusted p values*

|  | CN-eMCI (df = 1, 91) | | | CN-lMCI (df = 1, 74) | | | eMCI-lMCI (df = 1, 88) | | |
| --- | --- | --- | --- | --- | --- | --- | --- | --- | --- |
| Density | *F* | *p* | Adjusted *p* | *F* | *p* | Adjusted *p* | *F* | *p* | Adjusted *p* |
| 2.5 | 4.39 | .009 | .013 | 3.92 | .006 | .013 | 0.63 | .429 | .429 |
| 5 | 5.41 | .009 | .014 | 5.10 | .008 | .014 | 0.83 | .371 | .371 |
| 7.5 | 4.60 | .030 | .045 | 4.90 | .019 | .045 | 0.93 | .322 | .322 |
| 10 | 3.84 | .048 | .071 | 4.73 | .026 | .071 | 1.17 | .278 | .278 |
| 12.5 | 2.79 | .101 | .152 | 4.47 | .033 | .098 | 1.66 | .209 | .209 |

Table S9

*Left posterior hippocampal redundancy-memory regressions across densities with standardized beta and p values*

|  | Whole group | | CN | | eMCI | | lMCI | |
| --- | --- | --- | --- | --- | --- | --- | --- | --- |
| Density | ß | *p* | ß | *p* | ß | *p* | ß | *p* |
| 2.5 | 0.29 | .001 | 0.38 | .017 | 0.13 | .370 | -0.14 | .439 |
| 5 | 0.32 | < .001 | 0.37 | .022 | 0.12 | .380 | -0.01 | .971 |
| 7.5 | 0.30 | .001 | 0.33 | .043 | 0.17 | .230 | -0.05 | .784 |
| 10 | 0.30 | .001 | 0.33 | .041 | 0.20 | .160 | -0.04 | .832 |
| 12.5 | 0.29 | .001 | 0.36 | .025 | 0.19 | .188 | -0.12 | .495 |
| 15 | 0.29 | .001 | 0.38 | .020 | 0.16 | .258 | -0.13 | .459 |
| 17.5 | 0.27 | .002 | 0.38 | .021 | 0.18 | .210 | -0.17 | .355 |
| 20 | 0.28 | .002 | 0.39 | .017 | 0.19 | .179 | -0.14 | .442 |
| 22.5 | 0.28 | .002 | 0.37 | .024 | 0.22 | .133 | -0.15 | .400 |
| 25 | 0.28 | .002 | 0.37 | .028 | 0.21 | .135 | -0.14 | .442 |

Table S10

*Right posterior hippocampal redundancy-memory regressions across densities with standardized beta and p values*

|  | Whole group | | CN | | eMCI | | lMCI | |
| --- | --- | --- | --- | --- | --- | --- | --- | --- |
| Density | ß | *p* | ß | *p* | ß | *p* | ß | *p* |
| 2.5 | 0.05 | .608 | 0.02 | .912 | 0.07 | .608 | -0.25 | .136 |
| 5 | 0.11 | .230 | -0.05 | .784 | 0.18 | .215 | -0.12 | .482 |
| 7.5 | 0.10 | .279 | -0.11 | .520 | 0.19 | .173 | -0.13 | .473 |
| 10 | 0.11 | .225 | -0.11 | .523 | 0.22 | .124 | -0.11 | .537 |
| 12.5 | 0.16 | .078 | -0.04 | .786 | 0.25 | .071 | -0.09 | .617 |
| 15 | 0.17 | .061 | -0.03 | .871 | 0.29 | .035 | -0.12 | .492 |
| 17.5 | 0.17 | .055 | -0.02 | .886 | 0.32 | .022 | -0.14 | .413 |
| 20 | 0.18 | .039 | 0.02 | .904 | 0.29 | .038 | -0.12 | .498 |
| 22.5 | 0.21 | .017 | 0.02 | .894 | 0.33 | .016 | -0.11 | .541 |
| 25 | 0.23 | .009 | 0.09 | .600 | 0.34 | .015 | -0.10 | .582 |

Table S11

*Left posterior hippocampal redundancy-executive function regressions across densities*

|  | Whole group | | CN | | eMCI | | lMCI | |
| --- | --- | --- | --- | --- | --- | --- | --- | --- |
| Density | ß | *p* | ß | *p* | ß | *p* | ß | *p* |
| 2.5 | 0.13 | .142 | 0.21 | .197 | 0.01 | .943 | -0.27 | .123 |
| 5 | 0.12 | .187 | 0.15 | .353 | 0.05 | .700 | -0.25 | .180 |
| 7.5 | 0.10 | .267 | 0.14 | .387 | 0.06 | .678 | -0.22 | .211 |
| 10 | 0.09 | .297 | 0.14 | .403 | 0.09 | .529 | -0.24 | .172 |
| 12.5 | 0.09 | .316 | 0.18 | .271 | 0.06 | .657 | -0.28 | .119 |
| 15 | 0.08 | .359 | 0.17 | .321 | 0.08 | .580 | -0.28 | .116 |
| 17.5 | 0.08 | .369 | 0.19 | .252 | 0.10 | .495 | -0.30 | .088 |
| 20 | 0.07 | .435 | 0.18 | .288 | 0.09 | .554 | -0.28 | .110 |
| 22.5 | 0.08 | .387 | 0.20 | .239 | 0.10 | .492 | -0.30 | .088 |
| 25 | 0.07 | .465 | 0.18 | .288 | 0.09 | .544 | -0.28 | .108 |

Table S12

*Right posterior hippocampal redundancy-executive function regressions across densities*

|  | Whole group | | CN | | eMCI | | lMCI | |
| --- | --- | --- | --- | --- | --- | --- | --- | --- |
| Density | ß | *p* | ß | *p* | ß | *p* | ß | *p* |
| 2.5 | -0.11 | .234 | 0.02 | .924 | -0.15 | .269 | 0.36 | .031 |
| 5 | 0.02 | .854 | 0.10 | .529 | -0.07 | .644 | -0.33 | .054 |
| 7.5 | 0.02 | .865 | 0.11 | .495 | -0.03 | .839 | -0.31 | .066 |
| 10 | 0.02 | .831 | 0.14 | .395 | 0.01 | .946 | -0.32 | .062 |
| 12.5 | 0.03 | .714 | 0.18 | .265 | -0.00 | .995 | -0.32 | .062 |
| 15 | 0.03 | .762 | 0.12 | .454 | 0.03 | .827 | -0.27 | .119 |
| 17.5 | 0.03 | .720 | 0.12 | .466 | 0.03 | .827 | -0.23 | .185 |
| 20 | 0.03 | .748 | 0.11 | .512 | 0.03 | .838 | -0.22 | .211 |
| 22.5 | 0.04 | .650 | 0.08 | .633 | 0.07 | .615 | -0.20 | .247 |
| 25 | 0.04 | .632 | 0.06 | .732 | 0.07 | .638 | -0.16 | .353 |

Table S13

*Posterior hippocampal redundancy-cognition regressions for averaged density, using robust regression (Huber weighting) and Wald test for significance*

|  | *Left Posterior* | | | *Right Posterior* | | |
| --- | --- | --- | --- | --- | --- | --- |
|  | ß | *t* | *p* | ß | *t* | *p* |
| Memory | | | |  |  |  |
| Whole group | 0.19 | 3.02 | .003 | 0.14 | 2.27 | .025 |
| CN | 0.20 | 2.08 | .043 | 0.04 | 0.45 | .654 |
| eMCI | 0.12 | 1.42 | .161 | 0.19 | 2.16 | .034 |
| lMCI | -0.12 | 1.36 | .187 | -0.11 | 1.19 | .246 |
| Executive Function | | | |  |  |  |
| Whole group | 0.06 | 0.70 | .485 | 0.04 | 0.52 | .602 |
| CN | 0.13 | 0.82 | .410 | 0.10 | 0.62 | .532 |
| eMCI | 0.08 | 0.62 | .532 | 0.02 | 0.19 | .849 |
| lMCI | -0.31 | 1.89 | .069 | -0.19 | 1.13 | .269 |

Table S14

*Hippocampal degree omnibus test statistics across densities (df = 2, 126)*

|  | Left Anterior | | Right Anterior | | Left Posterior | | Right Posterior | |
| --- | --- | --- | --- | --- | --- | --- | --- | --- |
| Density | *F* | *p* | *F* | *p* | *F* | *p* | *F* | *p* |
| 2.5 | 0.21 | .820 | 0.43 | .656 | 1.87 | .155 | 0.42 | .663 |
| 5 | 0.01 | .994 | 0.49 | .618 | 1.97 | .145 | 0.72 | .497 |
| 7.5 | 0.30 | .741 | 0.38 | .680 | 3.07 | .046 | 1.33 | .269 |
| 10 | 0.93 | .401 | 0.68 | .513 | 2.83 | .060 | 1.18 | .316 |
| 12.5 | 1.14 | .335 | 0.74 | .483 | 2.53 | .083 | 0.65 | .532 |
| 15 | 1.70 | .183 | 0.89 | .428 | 2.70 | .073 | 0.39 | .670 |
| 17.5 | 1.32 | .268 | 0.90 | .409 | 2.75 | .065 | 0.55 | .576 |
| 20 | 0.93 | .403 | 0.67 | .503 | 2.42 | .087 | 1.00 | .375 |
| 22.5 | 0.82 | .439 | 0.78 | .462 | 2.45 | .086 | 1.33 | .264 |
| 25 | 1.18 | .306 | 0.85 | .429 | 2.18 | .115 | 1.60 | .204 |

*Note*: Follow-up tests of left posterior degree at density 7.5 revealed no significant group differences after correcting for multiple comparisons [CN-eMCI: *F*(1, 91) = 5.49, *p* = .061; CN-lMCI: *F*(1, 74) = 2.45, *p* = .175; eMCI-lMCI: *F*(1, 88) = 0.25, *p* = .623].

Table S15

*Hippocampal redundancy-white matter hyperintensities regressions for averaged density, using robust regression (Huber weighting) and Wald test for significance*

|  | ß | *t* | *p* |
| --- | --- | --- | --- |
| Left Anterior | -0.003 | 0.03 | .977 |
| Right Anterior | 0.177 | 1.65 | .100 |
| Left Posterior | 0.003 | 0.04 | .964 |
| Right Posterior | 0.079 | 0.92 | .357 |

Table S16

*Hippocampal redundancy-global efficiency regressions across densities collapsed across group*

|  | Left Anterior | | Right Anterior | | Left Posterior | | Right Posterior | |
| --- | --- | --- | --- | --- | --- | --- | --- | --- |
| Density | ß | *p* | ß | *p* | ß | *p* | ß | *p* |
| 2.5 | -0.03 | .736 | -0.02 | .859 | 0.06 | .523 | 0.14 | .123 |
| 5 | -0.09 | .285 | -0.08 | .369 | 0.07 | .423 | 0.04 | .657 |
| 7.5 | -0.11 | .231 | -0.05 | .603 | 0.04 | .652 | 0.11 | .205 |
| 10 | -0.13 | .150 | -0.11 | .234 | 0.08 | .385 | 0.16 | .067 |
| 12.5 | -0.16 | .071 | -0.12 | .182 | 0.07 | .424 | 0.11 | .195 |
| 15 | -0.20 | .024 | -0.12 | .192 | 0.05 | .547 | 0.11 | .219 |
| 17.5 | -0.20 | .024 | -0.11 | .210 | 0.04 | .646 | 0.05 | .542 |
| 20 | -0.18 | .035 | -0.08 | .383 | 0.04 | .640 | 0.02 | .823 |
| 22.5 | -0.16 | .071 | -0.06 | .507 | 0.00 | .996 | -0.05 | .591 |
| 25 | -0.11 | .215 | -0.02 | .817 | -0.03 | .768 | -0.11 | .213 |

Table S17

*Hippocampal redundancy-global efficiency regressions across densities within CN group*

|  | Left Anterior | | Right Anterior | | Left Posterior | | Right Posterior | |
| --- | --- | --- | --- | --- | --- | --- | --- | --- |
| Density | ß | *p* | ß | *p* | ß | *p* | ß | *p* |
| Avg. | -0.25 | .127 | 0.13 | .414 | -0.10 | .530 | -0.19 | .259 |
| 2.5 | 0.02 | .899 | 0.28 | .068 | 0.03 | .835 | -0.07 | .643 |
| 5 | 0.02 | .911 | 0.17 | .282 | -0.03 | .838 | -0.17 | .275 |
| 7.5 | -0.02 | .904 | 0.30 | .056 | -0.12 | .437 | -0.12 | .433 |
| 10 | -0.04 | .815 | 0.20 | .219 | -0.10 | .525 | -0.08 | .617 |
| 12.5 | -0.14 | .391 | 0.11 | .497 | -0.10 | .509 | -0.14 | .394 |
| 15 | -0.22 | .196 | 0.09 | .594 | -0.13 | .418 | -0.13 | .406 |
| 17.5 | -0.28 | .098 | 0.10 | .530 | -0.17 | .293 | -0.20 | .193 |
| 20 | -0.26 | .125 | 0.14 | .373 | -0.18 | .243 | -0.28 | .070 |
| 22.5 | -0.22 | .189 | 0.15 | .360 | -0.23 | .137 | -0.39 | .011 |
| 25 | -0.14 | .418 | 0.20 | .223 | -0.25 | .113 | -0.43 | .004 |

Table S18

*Hippocampal redundancy-global efficiency regressions across densities within eMCI group*

|  | Left Anterior | | Right Anterior | | Left Posterior | | Right Posterior | |
| --- | --- | --- | --- | --- | --- | --- | --- | --- |
| Density | ß | *p* | ß | *p* | ß | *p* | ß | *p* |
| Avg. | -0.11 | .431 | -0.11 | .429 | 0.32 | .019 | 0.14 | .306 |
| 2.5 | -0.01 | .922 | -0.23 | .102 | 0.33 | .018 | 0.26 | .058 |
| 5 | -0.10 | .496 | -0.25 | .081 | 0.41 | .003 | 0.31 | .027 |
| 7.5 | -0.12 | .396 | -0.21 | .132 | 0.37 | .007 | 0.32 | .022 |
| 10 | -0.18 | .211 | -0.27 | .050 | 0.32 | .020 | 0.28 | .044 |
| 12.5 | -0.18 | .215 | -0.28 | .046 | 0.29 | .037 | 0.22 | .124 |
| 15 | -0.20 | .168 | -0.27 | .056 | 0.25 | .068 | 0.18 | .207 |
| 17.5 | -0.17 | .260 | -0.26 | .067 | 0.27 | .051 | 0.14 | .340 |
| 20 | -0.15 | .296 | -0.20 | .161 | 0.30 | .034 | 0.12 | .396 |
| 22.5 | -0.14 | .357 | -0.18 | .219 | 0.26 | .061 | 0.09 | .535 |
| 25 | -0.06 | .671 | -0.14 | .325 | 0.26 | .060 | 0.06 | .679 |

Table S19

*Hippocampal redundancy-global efficiency regressions across densities within lMCI group*

|  | Left Anterior | | Right Anterior | | Left Posterior | | Right Posterior | |
| --- | --- | --- | --- | --- | --- | --- | --- | --- |
| Density | ß | *p* | ß | *p* | ß | *p* | ß | *p* |
| Avg. | 0.17 | .328 | -0.10 | .538 | 0.05 | .763 | 0.25 | .132 |
| 2.5 | 0.16 | .402 | -0.09 | .612 | 0.04 | .808 | 0.29 | .105 |
| 5 | -0.08 | .673 | -0.06 | .729 | 0.12 | .493 | 0.26 | .142 |
| 7.5 | 0.03 | .861 | -0.07 | .664 | 0.05 | .797 | 0.23 | .190 |
| 10 | 0.07 | .697 | -0.12 | .497 | 0.06 | .747 | 0.25 | .146 |
| 12.5 | 0.14 | .416 | -0.10 | .570 | 0.05 | .777 | 0.25 | .153 |
| 15 | 0.17 | .349 | -0.08 | .660 | 0.06 | .734 | 0.31 | .077 |
| 17.5 | 0.20 | .263 | -0.08 | .643 | 0.06 | .726 | 0.29 | .100 |
| 20 | 0.20 | .252 | -0.08 | .663 | 0.13 | .461 | 0.30 | .085 |
| 22.5 | 0.24 | .167 | -0.01 | .942 | 0.13 | .451 | 0.28 | .106 |
| 25 | 0.23 | .190 | 0.00 | .991 | 0.15 | .387 | 0.26 | .138 |

Table S20

*Hippocampal redundancy-global efficiency regressions for averaged density, using robust regression (Huber weighting) and Wald test for significance*

|  | Left Anterior | | | Right Anterior | | | Left Posterior | | | Right Posterior | | |
| --- | --- | --- | --- | --- | --- | --- | --- | --- | --- | --- | --- | --- |
|  | ß | *t* | *p* | ß | *t* | *p* | ß | *t* | *p* | ß | *t* | *p* |
| Whole group | -0.16 | 1.89 | .064 | -0.10 | 1.17 | .256 | 0.12 | 1.39 | .208 | 0.07 | 0.84 | .418 |
| CN | -0.28 | 1.72 | .091 | 0.05 | 0.33 | .755 | -0.15 | 0.95 | .346 | -0.26 | 1.62 | .112 |
| eMCI | -0.07 | 0.47 | .641 | -0.13 | 0.92 | .378 | 0.32 | 2.37 | .021 | 0.16 | 1.19 | .233 |
| lMCI | 0.14 | 1.10 | .276 | -0.12 | 0.90 | .364 | -0.06 | 0.48 | .637 | 0.17 | 1.30 | .202 |

**Supplemental References**

1 Joshi A.D. *et al.* Performance characteristics of amyloid PET with florbetapir F 18 in patients with Alzheimer’s disease and cognitively normal subjects. *J Nucl Med*. **53**, 378–384 (2012).

2 Clark C.M. *et al.* Use of florbetapir-PET for imaging β-amyloid pathology. *JAMA - J Am Med Assoc*. **305**, 275–283 (2011).

3 Gibbons L.E. *et al.* Composite measures of executive function and memory: ADNI_EF and ADNI_Mem. Alzheimer’s Disease Neuroimaging Initiative. https://adni.bitbucket.io/reference/docs/UWNPSYCHSUM/ADNI_Methods_UWNPSYCHSUM.pdf (2015).
